# Simulation-Based Training Tools for Cleft Lip Repair: A Systematic Review

**DOI:** 10.3390/jcm15207911

**Published:** 2026-10-13

**Authors:** Aleksander Jaworski, Katarzyna Ochman, Kamil Szemik, Dominika Kaczyńska, Oliwia Barnaś, Aleksandra Gaweł, Wiktoria Nowojewska, Zofia Pankowska, Marta Młynek, Anna Kożuch, Jakub Kufel, Ireneusz Łątkowski, Aneta Drozdowska, Piotr Drozdowski

**Affiliations:** 1 Department of Plastic Surgery, St. John Paul II Specialist Medical Centre, 57-320 Polanica-Zdrój, Poland; 2 SudetiaMed Medical Center, 57-300 Kłodzko, Poland; 3 Students’ Scientific Association of Computer Analysis and Artificial Intelligence at the Department of Radiology and Nuclear Medicine, Medical University of Silesia, 40-752 Katowice, Poland; 4 Department of Dermatology, School of Medicine in Katowice, Medical University of Silesia, 40-027 Katowice, Poland; 5 Department of Radiology and Nuclear Medicine, Medical University of Silesia, 40-752 Katowice, Poland

**Keywords:** cleft lip repair, simulation-based training, surgical simulation, surgical education, surgical training models

## Abstract

**Background**: Simulation may supplement operative exposure in cleft lip surgery, but the strength and clinical relevance of evidence remain uncertain. This systematic review evaluated cleft lip repair simulators, focusing on characteristics, validity evidence, educational effectiveness, and implementation. **Methods**: PubMed, Scopus, Web of Science, and Embase were searched on 1 August 2026. Eligible studies were original English-language studies published in 2016–2026 evaluating cleft lip repair simulators for training or assessment. Non-original reports and studies without an eligible training or assessment component were excluded. Two reviewers independently selected studies and extracted data. Validity evidence was assessed using Messick’s framework, educational effectiveness using an adapted McGaghie framework, and methodological quality using the Mixed Methods Appraisal Tool (MMAT) 2018. Findings were synthesized narratively. The protocol was submitted to PROSPERO on 5 August 2026, and registered on 11 August 2026 (CRD420261469105). **Results**: Twenty-four publications were included. Two publications (8.3%) were categorized as educational effectiveness not evaluated, 14 (58.3%) as McGaghie Level 1, and eight (33.3%) as Level 2; none reached Levels 3–5. Eight publications demonstrated objectively measured improvement in knowledge or performance following an educational intervention or repeated simulation. Response Process validity evidence was identified in 19 publications (79.2%), whereas Content-related validity evidence was identified in 13 publications (54.2%). Internal Structure and Relations to Other Variables were less frequently supported, and Consequences evidence was absent. Evidence was limited by small samples, heterogeneous designs, and frequent reliance on subjective outcomes. **Conclusions**: Cleft lip simulation can produce measurable educational benefits, but evidence of transfer to clinical performance or patient outcomes is lacking. Future studies should prioritize objective assessment, stronger validity and reliability evidence, accessible task-specific models, and evaluation of clinical transfer. No external funding was received.

## 1. Introduction

Cleft lip, occurring either in isolation or in association with cleft palate, is one of the most common congenital craniofacial malformations, with a global incidence estimated at 0.3 cases per 1000 live births for isolated cleft lip and 0.45 per 1000 for cleft lip associated with cleft palate [1]. Embryologically, this defect results from impaired fusion of the maxillary processes with the medial nasal processes during early embryonic development [2,3]. Clinically, the condition presents a broad phenotypic spectrum, ranging from a microform notch of the vermilion and subepithelial discontinuity of the orbicularis oris muscle to a complete unilateral or bilateral cleft extending through the full height of the lip to the base of the nose [4,5].

Beyond its anatomical consequences, cleft lip may affect patients’ psychosocial functioning, including their self-esteem [6]. The complexity of these issues supports a multidisciplinary approach to treatment, in which primary surgical reconstruction represents an important component [7,8].

Surgical correction of cleft lip (cheiloplasty) is a complex procedure within pediatric plastic and reconstructive surgery. The aims of the operation include restoring continuity of the orbicularis oris muscle, as well as re-establishing facial symmetry, the contour of the vermilion, and the anatomy of the nasolabial complex [9]. Performing this procedure in an infant requires precise identification of preoperative anatomical landmarks and careful planning because of the small size of the structures involved [10]. Potential inaccuracies in restoring muscular continuity or planning the incisions may lead to facial asymmetry, distortion of the nasal ala, and, in some cases, the need for secondary surgical correction [9,11].

The traditional model of surgical education, based on training in the operating room, is associated with limitations related to factors such as training time, procedure-specific constraints, and the need to ensure patient safety [12,13,14]. These factors may hinder surgical trainees from achieving operative autonomy and limit opportunities for repeated practice of technical skills in clinical settings [12,14]. Simulators provide a controlled environment in which surgical trainees can repeatedly practice incision planning and tissue dissection without time pressure or risk to the patient [15,16]. The opportunity to perform the procedure repeatedly and to safely make and correct errors may support the development of technical skills and increase trainees’ confidence [16,17]. 

Simulators may be particularly useful in settings where access to specialized surgical training is limited. Increasing the availability of a trained surgical workforce remains an important challenge in global health, particularly in low- and middle-income countries [18]. In this context, low-cost and readily available training models may complement traditional operative training [19].

Contemporary simulators for cheiloplasty vary in their level of fidelity and fabrication methods. These include biological ex vivo models, such as prepared porcine snouts, which, owing to their natural layered structure, provide realistic haptic feedback at relatively low cost [19]. Advances in computer-aided design and three-dimensional printing technology have also enabled the development of physical, multilayered models that reproduce key anatomical features of the lip and nasolabial complex [15,20,21]. The use of silicones and polymers enables the properties of individual model layers to be tailored in order to reproduce tissue behavior during dissection and suturing [20,21].

The evaluation of these tools may include validation studies using objective technical skills assessment scales, such as Objective Structured Assessment of Technical Skills (OSATS) [22,23]. Across studies, users also evaluated the realism of the models, their training utility, and the effect of training on confidence in performing the procedure [17,20,24].

Studies from other areas of surgery suggest potential benefits of simulation-based training, including improvements in technical skills and the possibility of transferring these skills to real-world operative settings [25]. For example, the systematic review by Lastoria et al. included 116 studies on simulation tools used in areas including hand, breast, facial, cleft, and burn surgery, as well as general plastic surgery skills training, while highlighting the limited evidence regarding the transfer of acquired skills to clinical practice and the need for further validation studies [12]. 

Within the more specialized field of craniofacial surgery, reviews have examined the use of simulation in cleft surgery [26], while systematic reviews have also specifically focused on models for cleft palate repair [27]. To date, no systematic review has been identified that focuses exclusively on simulators used for surgical cleft lip repair.

The aim of this study is to conduct a systematic review of contemporary simulation-based training tools used in cleft lip repair, focusing on developments published over the past decade, and to synthesize the available evidence regarding their design, realism, validation, usability, and educational effectiveness.

## 2. Materials and Methods

### 2.1. Search Strategy

The review protocol was developed a priori and first submitted to PROSPERO on 5 August 2026. The record was registered on 11 August 2026 (registration number: CRD420261469105). Registration was completed after study screening, which took place between 5 and 8 August 2026, and during data extraction, which took place between 8 and 22 August 2026. The complete protocol is available in Supplementary File S1. A systematic literature search was performed to identify original studies evaluating simulation-based training, education, or assessment tools used in cleft lip surgery. The review was reported in accordance with the PRISMA 2020 statement [28]. The completed PRISMA 2020 and PRISMA 2020 for Abstracts checklists are provided together in Supplementary File S2. The search strategy was developed according to the PICO framework and adapted to the educational and simulation-based context of the review, provided in Supplementary File S3. Search terms covered the concepts of cleft lip surgical repair and simulation-based training, including simulators, task trainers, surgical models, and related simulation modalities. Controlled vocabulary (MeSH and Emtree terms) and free-text keywords were combined using Boolean operators.

Searches were conducted on 1 August 2026, in PubMed, Scopus, Web of Science, and Embase. During revision, the PubMed search was re-run on 23 September 2026 to standardize the publication-date limits to 1 January 2016–1 August 2026. This retrieved five additional records, all of which were duplicates of records already identified through the other databases; therefore, the number of unique records screened and the set of included publications remained unchanged. The PRISMA flow diagram reports the updated PubMed retrieval count. The search was restricted to publications from 2016 to 2026 to capture contemporary simulation technologies and educational approaches in cleft lip surgery. This publication window was selected to reflect the rapid development of modern simulation modalities, including three-dimensional printing, digital simulation, virtual and augmented reality, and high-fidelity physical training models. Date restrictions were applied at the database-search stage, whereas no language restriction was applied during database searching; the English-language restriction was applied during study screening according to the predefined eligibility criteria. Backward citation searching of the reference lists of included studies and previous reviews, as well as forward citation searching, was not performed. Database-specific filters were applied as documented in the complete search strategies provided in the Supplementary File S4.

### 2.2. Eligibility Criteria

Eligible studies were original peer-reviewed research articles evaluating simulators, task trainers, training models, or other tools intended for education, surgical training, or assessment in cleft lip repair. Assessment-focused studies were eligible when the simulator or simulation-based tool itself was evaluated or validated as a training or assessment tool. Studies were excluded when the simulator served solely as a standardized research or assessment platform to evaluate a separate assessment instrument, trainee performance patterns, or another research question, without evaluating the simulator itself or its utility for education, training, or assessment. Accordingly, studies in which an existing simulator was used only to develop or evaluate a separate assessment instrument or to characterize trainee performance were excluded, whereas studies evaluating the simulator itself as a tool for competency assessment were eligible. Studies focused on anatomical teaching were eligible only when anatomical learning was integrated with, or directly linked to, the practice or assessment of a defined cleft lip surgical procedure or operative step. Studies limited to anatomical demonstration, visualization, or theoretical knowledge acquisition without a procedure-specific surgical training or assessment component were excluded. Eligible studies could address unilateral or bilateral cleft lip, primary cleft lip repair, cheiloplasty, or a clearly defined stage of the procedure. Physical, synthetic, silicone, three-dimensionally printed, biological, animal-based, cadaveric, virtual, haptic, and hybrid simulation modalities were eligible. Studies were required to report data on the development, construction, evaluation, validity, usability, educational effectiveness, or assessment utility of the simulation-based tool. Studies addressing both cleft lip and cleft palate were included only when the cleft lip component was clearly described and the relevant data could be extracted separately. Exclusion criteria included focusing exclusively on cleft palate, alveolar clefts, alveolar bone grafting, or other craniofacial procedures without a clearly identifiable cleft lip repair component; general suturing, microsurgical, plastic surgery, or reconstructive surgery models without a specific application to cleft lip repair; or preoperative planning, anatomical visualization, treatment-outcome simulation, intraoperative navigation, patient counseling, or clinical treatment without an educational or training component. Studies describing only theoretical concepts or proposed training tools without construction, implementation, practical use, or evaluation were also excluded. Case reports, systematic reviews, scoping reviews, meta-analyses, narrative reviews, protocols, guidelines, commentaries, letters to the editor, expert opinions, and conference abstracts without an available full-text publication were excluded. Only full-text articles published in English between 2016 and 2026 were eligible. Assessment-focused studies were distinguished according to whether they evaluated the simulator’s utility for competency assessment or used it solely as a platform to investigate a separate assessment instrument or trainee performance. Study-specific reasons for full-text exclusion, including the assessment-platform and anatomy-education reports, are provided in Supplementary File S5.

### 2.3. Study Selection and Data Extraction

**Figure 1:**
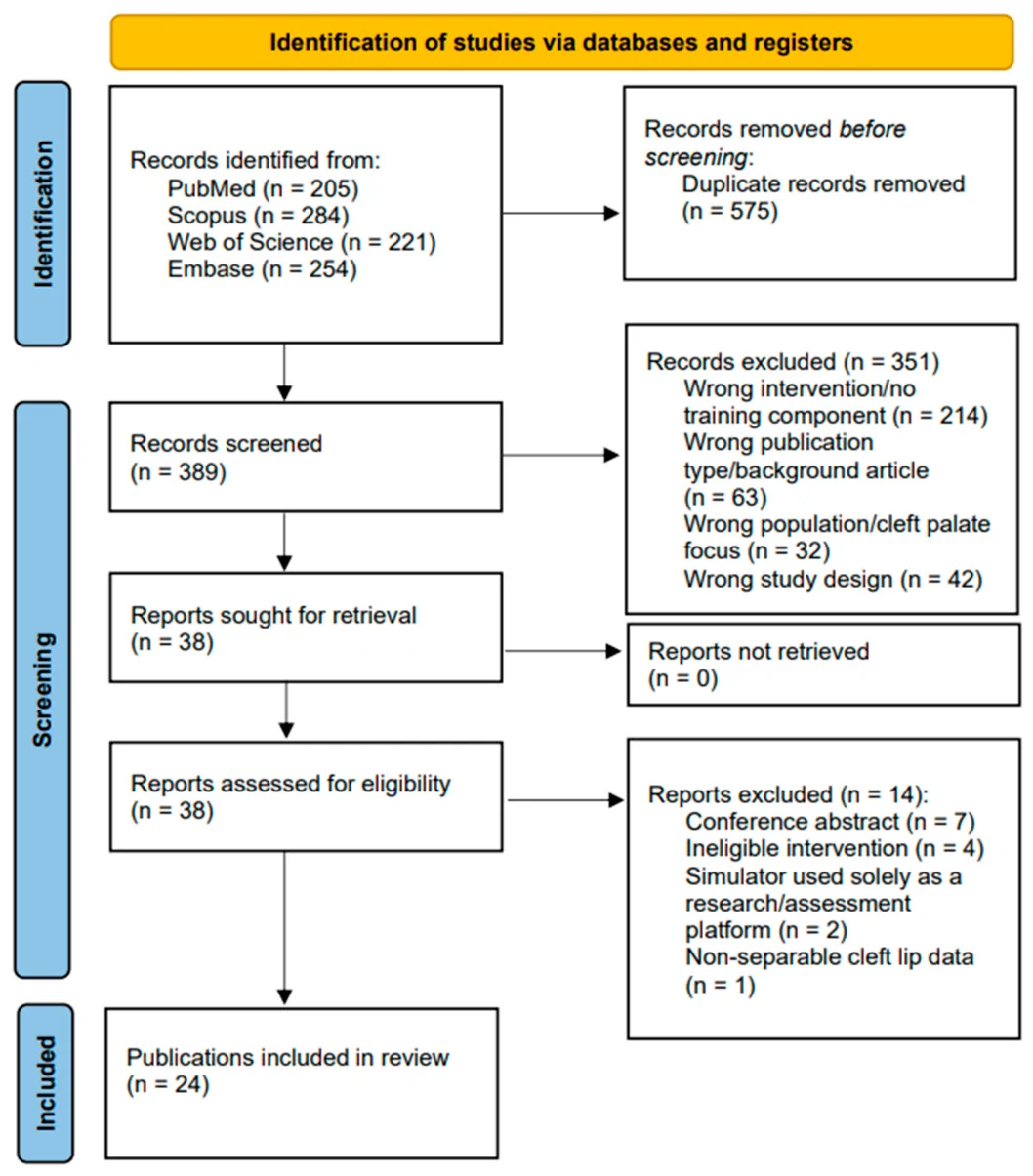
PRISMA flow diagram of the search and selection process.

All records retrieved from the database searches were imported into Rayyan, which was used for duplicate management and study screening [29]. Title and abstract screening was performed independently by a sixth-year and a third-year medical student, both with prior experience in systematic review methodology and literature screening. Full-text eligibility assessment was performed independently by a sixth-year medical student with substantial prior experience in systematic reviews and a plastic surgery resident. Disagreements during the study selection process were adjudicated by a specialist in plastic surgery. The study selection process is illustrated in the PRISMA 2020 flow diagram presented in Figure 1. Agreement between reviewers during title and abstract screening was assessed using Cohen’s kappa (κ), with strength of agreement interpreted according to the thresholds reported by Belur et al. [30]. Title and abstract screening and full-text eligibility assessment were conducted between 5 and 8 August 2026.

Data extraction was performed independently by a sixth-year medical student and a postgraduate medical intern, under the supervision of the plastic surgery resident. Data extraction was conducted between 8 and 22 August 2026. Any discrepancies during data extraction were resolved through discussion, and unresolved disagreements were adjudicated by a specialist in plastic surgery. Extracted data included study and participant characteristics, simulator and training characteristics, assessment methods, validity-related evidence, educational outcomes, implementation-related variables, and information on study limitations, funding, and conflicts of interest. Where studies evaluated multiple simulators, interventions, or study groups, outcomes were recorded separately when reported independently. Information not explicitly reported in the original publication was recorded as not reported and was not inferred. The complete publication-level data extraction table is provided in Supplementary File S5. Reasons for exclusion at the full-text stage were documented and are presented in the same Supplementary File, together with the list of included publications and full-text reports excluded after eligibility assessment.

### 2.4. Data Synthesis and Evidence Classification

Due to substantial heterogeneity in simulation modalities, participant characteristics, training protocols, and reported outcomes, a quantitative meta-analysis was not considered appropriate. Therefore, the findings were synthesized narratively, with studies compared according to simulator characteristics, training approaches, assessment methods, implementation characteristics, and educational outcomes.

The Messick validity framework and the adapted McGaghie translational outcomes framework were not specified by name in the registered protocol and were introduced subsequently as methodological refinements to structure the synthesis of validity-related evidence and educational effectiveness, respectively. Both frameworks were selected, and their initial operational criteria were established, before the original independent publication-level ratings were assigned. During manuscript revision, the operational criteria were clarified and the final judgments were re-examined. During minor revision, both reviewers independently reassessed all included publications using the final operational criteria for Messick validity evidence and adapted McGaghie educational effectiveness. Percentage agreement and unweighted Cohen’s κ were calculated from these updated independent ratings before consensus adjudication. Disagreements were subsequently resolved through discussion to establish the final classifications. Messick’s framework was selected to provide a structured evaluation of evidence supporting the interpretation of simulator- and assessment-derived observations or scores, whereas the adapted McGaghie framework was used to distinguish learner-level outcomes from objective educational effects and higher levels of clinical translation.

Validity evidence supporting the interpretation of simulator- and assessment-derived observations or scores was evaluated according to Messick’s framework across five domains: Content, Response Process, Internal Structure, Relations to Other Variables, and Consequences [31]. Each domain was independently rated using a four-category rating scale adapted from Beckman et al. (N, 0, 1, or 2), with domain-specific criteria operationalized for the purposes of this review [32]. Domain-specific operational criteria were defined before publication-level ratings were assigned to distinguish absent or non-assessable evidence (N), evidence not supporting the intended interpretation (0), limited or methodologically incomplete supporting evidence (1), and strong, direct, and methodologically robust supporting evidence (2). The criteria were tailored separately to Content, Response Process, Internal Structure, Relations to Other Variables, and Consequences, and are presented in detail in Supplementary File S6, Table S1. N indicated that relevant evidence was not reported or could not be assessed, 0 indicated that the domain was assessed but the findings did not support the intended interpretation, 1 indicated limited, indirect, partial, or methodologically incomplete supporting evidence, and 2 indicated strong, direct, and methodologically robust supporting evidence. Within the N category, we distinguished between domains that were not assessed in the original study and domains for which reporting was insufficient to determine whether an assessment had been performed. In contrast, a rating of 0 indicated that the domain was explicitly assessed but the findings did not support the intended interpretation. Domain ratings were considered independently and were not combined into an overall score. Ratings were based exclusively on evidence explicitly reported in the included studies.

Anatomical realism, procedural fidelity, perceived usefulness, satisfaction, expert teaching, and the organization of training were not considered evidence of assessment validity by themselves unless they directly supported the intended interpretation of a defined simulator- or assessment-derived score or observation. Given the lower initial inter-reviewer agreement for the Content domain, all final Content-domain judgments were re-examined against these clarified operational criteria. Consequences evidence concerns the intended and unintended effects of interpreting and using assessment results, including their influence on educational or competency decisions and any associated benefits or harms. A proficiency threshold, pass/fail standard, or progression criterion may be relevant to such evidence, but its presence or absence alone does not establish whether the Consequences domain is supported [31].

Educational effectiveness was classified using an adapted five-level framework based on McGaghie’s translational outcomes framework [33]. The adaptation primarily separated subjective learner reactions and self-reported outcomes from objectively demonstrated educational outcomes occurring within the educational or simulated setting. Publications that did not report learner-reaction or learning outcomes were categorized as “educational effectiveness not evaluated” and were not assigned a McGaghie level. For publications in which educational effectiveness was evaluated, each study was assigned the highest level demonstrated by its reported outcomes: Level 1, learner reaction or other subjective outcomes, including perceived usefulness, satisfaction, realism, self-reported knowledge, confidence, or technical ability; these outcomes were classified as Level 1 because they reflected learner reaction or perceived educational benefit rather than objectively demonstrated learning or performance. Level 2 required objectively demonstrated improvement in knowledge or technical performance following an educational intervention or repeated simulation, including retention of objectively measured gains within an educational or simulated setting. Performance discrimination based solely on prior training or experience was treated as assessment or validity evidence and was not considered sufficient for Level 2 classification. Level 3 represented transfer of acquired skills to independently assessed clinical performance; Level 4, improvement in patient-level outcomes; and Level 5, organizational, healthcare-system, or population-level effects. Improvements or retention demonstrated exclusively within a simulated environment were classified as Level 2 and were not considered evidence of clinical transfer. Messick domain ratings and McGaghie classifications were independently assigned by two reviewers. Disagreements between reviewers were subsequently resolved through consensus adjudication. Inter-reviewer agreement before consensus adjudication was assessed using percentage agreement and Cohen’s kappa (κ), separately for each Messick domain and for the McGaghie classification. Publication-level independent reviewer ratings, final consensus ratings, and the corresponding rationale for the Messick validity domains and McGaghie educational effectiveness classification are provided in Supplementary File S6.

### 2.5. Methodological Quality Assessment

Methodological quality of the included publications was assessed using the Mixed Methods Appraisal Tool (MMAT), version 2018 [34]. Publications were classified according to their methodological design and evaluated using the corresponding MMAT criteria. Two screening questions were applied to all studies, followed by five design-specific criteria rated as “Yes”, “No”, or “Can’t tell”. For mixed methods studies, the qualitative and quantitative components were additionally appraised separately using the relevant MMAT criteria, followed by assessment of the mixed methods-specific criteria. No overall numerical score was calculated. Methodological quality assessment was performed independently by two reviewers. Disagreements were resolved through discussion, with adjudication by a third reviewer if consensus could not be reached. The registered protocol initially specified the use of design-specific critical appraisal tools, including JBI checklists where applicable. This approach was subsequently amended to use MMAT 2018 in order to provide a consistent methodological appraisal framework across the heterogeneous study designs included in the review. The amendment was reflected in the updated PROSPERO record (version 1.1), published on 12 September 2026, and was implemented before the formal methodological quality assessment of the included studies. No included study had undergone formal methodological appraisal using the originally planned design-specific tools before this amendment. The complete methodological quality assessment is provided in Supplementary File S7. Generative artificial intelligence (AI) tools, including ChatGPT (OpenAI; GPT-5.6 Thinking and GPT-6.0 Astra), were used to assist in study design, language editing, refinement of methodological wording, and organization and presentation of the review findings. They were not used to independently conduct the literature search, study screening, data extraction, methodological quality assessment, or final evidence classification. All AI-assisted outputs were critically reviewed and verified by the authors, and all final methodological, analytical, and interpretative decisions remained the responsibility of the authors.

## 3. Results

### 3.1. Characteristics of Included Publications

Twenty-four publications evaluating simulation-based education, training, simulator development, or simulator-based assessment in cleft lip surgery met the inclusion criteria. Inter-reviewer agreement during title and abstract screening was moderate (Cohen’s κ = 0.594) [30]. The included publications comprised simulator-development and feasibility studies, expert evaluations, prospective single-group educational studies, comparative educational studies, and randomized controlled trials. Participant populations ranged from undergraduate medical and dental students to plastic surgery and otolaryngology residents, craniofacial and pediatric otolaryngology fellows, and experienced cleft surgeons.

The evaluated simulation modalities comprised ex vivo biological models, silicone- and polymer-based physical simulators, three-dimensional printed models, multilayer high-fidelity simulators, stone models for surgical marking, and web-based digital simulation platforms. Publications addressed unilateral cleft lip repair, bilateral cleft lip repair, or both. Simulated tasks included anatomical orientation, surgical marking, incision planning, tissue dissection, orbicularis oris reconstruction, multilayer closure, and, in high-fidelity models designed for complete repair, primary rhinoplasty and nasal reconstruction.

Several publications arose from related simulator research programs and did not necessarily represent independent participant samples. Full-text comparison confirmed that the Rogers-Vizena competency-assessment and retention publications involved the same underlying cohort of 26 residents and fellows; in the later repeated-simulation study, 21 of the original 26 participants returned for the approximately three-month follow-up assessment [23,35]. The two Kantar publications involving 13 plastic surgery residents originated from the same institutional research program and may have included overlapping participants, although complete cohort identity could not be established from the published reports [36,37]. Similarly, participant overlap between the bilateral and unilateral Lutz publications could not be excluded [19,38]. Accordingly, numerical summaries in this review are reported as publication counts rather than counts of independent participant cohorts, and a single total number of unique participants was not calculated.

Reported outcomes included simulator development, anatomical fidelity, haptic properties, perceived educational value, knowledge acquisition, confidence, technical performance, comparison of educational modalities, competency assessment, and skill retention. Training interventions ranged from single simulator encounters to multimodal curricula incorporating didactic instruction, operative video material, supervised hands-on practice, feedback, and structured debriefing. Where simulation formed part of a multimodal educational intervention and the effects of individual components could not be separated, observed outcomes were attributed to the educational program as delivered rather than to the simulator alone. The key characteristics of all included publications are summarized in Table 1. Detailed publication-level characteristics are provided in Supplementary File S5.

### 3.2. Simulator Development, Realism, and Feasibility

Simulator-development publications primarily assessed anatomical fidelity, haptic properties, procedural feasibility, reproducibility of operative steps, and perceived training value. These characteristics were summarized separately from validity evidence supporting interpretation of a specific assessment score or observation.

Zaga-Galante et al. evaluated a high-fidelity bilateral cleft lip simulator by 11 experienced cleft surgeons. Mean ratings were 3.7/5 for realism and anatomical accuracy and 4.6/5 for training value. The prolabium position and orientation received the highest feature-specific score, whereas mucosal suturing received the lowest [39]. In Riedle et al., 83% of participants completed the simulated cleft repair and 100% reported perceived improvement in surgical technique; no objectively measured performance gain was reported [42].

Reighard et al. developed a low-cost high-fidelity model using computer-aided design and three-dimensional printing. Five cleft surgeons evaluated the simulator with a 22-item cognitive task analysis instrument, and three experts independently reviewed the assessment instrument [20]. Podolsky et al. evaluated an end-to-end unilateral simulator that permitted surgical marking, incision, tissue dissection, orbicularis repair, multilayer closure, and primary rhinoplasty [15]. Rogers-Vizena et al. evaluated a synthetic unilateral cleft lip and alveolus simulator in three cleft surgeons using three-dimensional pre- and postoperative scans and compared simulator-derived nasolabial changes with patient-derived patterns [21].

Teuber Lobos et al. explicitly evaluated face and content validity using a predefined Likert-based questionnaire completed by six cleft surgeons and ten trainees after simulated procedures. The questionnaire addressed anatomical appearance, tissue properties, haptic characteristics, procedural realism, and perceived educational utility [45].

### 3.3. Subjective Educational Outcomes

Fourteen publications were classified as adapted McGaghie Level 1 because their highest educational outcome consisted of learner reaction, perceived usefulness, satisfaction, realism, confidence, or self-reported knowledge/technical ability [15,17,19,20,24,36,38,39,42,43,44,45,49,50]. These endpoints were not treated as evidence of objectively demonstrated technical competence.

Khoo et al. reported a 53.5% increase in total self-assessed procedural confidence after a high-fidelity workshop in 11 plastic surgery trainees [17]. Chang et al. reported significant increases in 15 of 16 self-rated skill domains after a multicenter simulation course [24], while Michaels et al. found increased self-reported procedural expertise and confidence after a national virtual dissection course [49]. Participants using unilateral and bilateral porcine models also reported improved understanding, theoretical knowledge, and practical skills [19,38].

Saldanha et al. demonstrated significant changes in self-assessed knowledge/skill after a multimodal bootcamp curriculum [44]. Objective anthropometric measurements were also obtained, and training level was associated with some symmetry measures; however, these cross-sectional associations did not demonstrate objective improvement attributable to the intervention and were therefore not counted as objective educational gain. Stanek et al. reported self-assessed improvement with both reduced- and high-complexity simulators, but the between-group difference was not statistically significant (3.7 ± 2.2 versus 2.7 ± 1.9; *p* = 0.09; adjusted RR 1.30, 95% CI 0.96–1.77) [50].

### 3.4. Objective Knowledge and Technical Performance

Eight publications demonstrated objectively measured improvement in knowledge or performance following an educational intervention or repeated simulation and were classified as adapted McGaghie Level 2 [35,37,40,41,46,47,48,51]. To avoid conflating different strengths of evidence, these publications were considered according to the type of inference supported: direct comparative educational effects, objective improvement without an adequate between-group comparison, learning-curve effects, and retention of improvement.

Three publications provided direct comparative evidence favoring simulator-assisted education. Wang et al. randomized 40 senior stomatology students to three-dimensional simulator-assisted problem-based learning or conventional problem-based learning and reported higher post-training theoretical and operative scores in the simulator-assisted group [41]. Chen et al. randomized 53 undergraduate dental students to simulator-assisted or conventional teaching and reported a higher post-intervention knowledge score in the simulator-assisted group [47]. Plana et al. randomized 35 first-year medical students to 20 min of digital simulation or textbook-based learning and demonstrated significantly greater improvement in cleft lip marking performance with digital simulation (*p* < 0.001) [51].

Plana et al. assessed cleft lip marking performance using a 10-item binary scoring system with a total possible score of 0–10 points; higher scores indicated better marking performance. The Plana publication contains internally discrepant numerical estimates between the abstract and the main-text Results. The abstract reports a mean improvement of 5.81 ± 2.01 points in the digital-simulation group versus 2.68 ± 1.49 points in the textbook group, whereas the main-text Results report corresponding improvements of 5.17 ± 1.86 and 2.11 ± 1.27 points (*p* < 0.001 for the between-group comparison in both reports). The main-text Results also report comparable baseline scores between groups (0.57 ± 0.69 versus 0.44 ± 0.61; *p* = 0.56). Because the direction and statistical significance of the comparative effect are concordant but the numerical estimates differ, both sets of values were retained in Supplementary File S5 with an explicit discrepancy note rather than selecting one estimate as definitive. Intrarater ICCs were 0.94 and 0.95 for rater 1 and 0.60 and 0.92 for rater 2 for pre- and post-intervention ratings, respectively; interrater ICC increased from 0.78 before the intervention to 0.96 after the intervention. Confidence intervals for these ICC estimates were not reported in the article [51].

Kantar et al. randomized 13 plastic surgery residents to digital simulation or textbook-based learning. Surgical knowledge was scored from 0 to 45 points. The reported maximum scores were 10 points for marking performance, 20 points for modified OSATS surgical performance, and 50 points for procedural confidence; higher scores indicated better performance or greater confidence. Within the digital-simulation group, surgical knowledge increased from 33.5 ± 3.7 to 40.3 ± 4.4 (*p* = 0.03), marking performance from 2.9 ± 3.1 to 8.0 ± 2.5 (*p* = 0.03), and simulated surgical performance from 8.2 ± 2.3 to 12.3 ± 2.5 (*p* = 0.04); procedural confidence also increased from 14.7 ± 2.3 to 24.0 ± 7.0 (*p* = 0.03). Interrater ICCs for marking performance were 0.97 (95% CI, 0.89–0.99) before and 0.96 (95% CI, 0.87–0.99) after the intervention. Corresponding ICCs for simulated surgical performance were 0.83 (95% CI, 0.45–0.95) and 0.81 (95% CI, 0.37–0.94), with the wider intervals indicating greater uncertainty in the reliability of surgical performance ratings [37]. The textbook group did not demonstrate significant pre/post improvement; however, this alone does not establish a statistically significant between-group difference in change. Accordingly, the study was interpreted as demonstrating objective improvement following the digital-simulation intervention, without claiming superiority over textbook-based learning in the absence of an adequate direct between-group change analysis.

Three additional publications demonstrated objective improvement without a concurrent comparator. Volk et al. reported improvement in objective examination knowledge after use of the Smile Train Virtual Surgery Simulator in 16 plastic surgery trainees; scores increased from 33.1% to 64.4% among junior residents and from 46.9% to 70.8% among senior residents [48]. Vogl et al. reported improvement in procedure-related anatomy tasks following training with unilateral and bilateral ex vivo cleft lip models, with mean scores increasing from 4.44 to 6.07 for unilateral cleft anatomy and from 2.31 to 3.44 for bilateral cleft anatomy; both tasks had a maximum score of 7 points [40].

Kaimi et al. used a 13-item cleft lip-specific assessment scale, with each item rated from 1 to 5, giving a theoretical total range of 13–65 points. The global rating scale presented in the Methods section and Table 2 of the original publication by Kaimi et al. [46] comprised six items rated from 1 to 5 after omission of the “use of assistants” item, implying a theoretical total range of 6–30 points. However, Table 6 in the original publication by Kaimi et al. [46] labels the global score maximum as 35. This inconsistency was retained as a reporting limitation rather than resolved by assuming either maximum to be definitive. Kaimi et al. demonstrated a progressive learning curve across three sequential simulated repairs, with mean cleft lip-specific scores increasing from 28.5 ± 5.85 to 36.3 ± 9.12 and 43.5 ± 9.02 (*p* = 0.015), while global scores increased from 12.9 ± 3.77 to 17.3 ± 3.38 and 21.1 ± 4.73 (*p* = 0.027) [46]. Because these studies lacked a concurrent comparator, the observed improvements cannot be separated from potential practice effects, repeated testing, or concurrent educational exposure.

Rogers-Vizena et al. provided the only dedicated retention study. Twenty-six residents and fellows completed the first two simulation sessions, and 21 returned for the approximately three-month follow-up, corresponding to 19.2% attrition. Objective OSATS and UCLR performance improved after the initial simulation-plus-structured-debriefing cycle and remained above baseline at follow-up [35]. Because follow-up was incomplete, the retention finding was interpreted as evidence of maintained improvement among participants who returned rather than as complete-cohort evidence, and the attrition was explicitly considered in the methodological appraisal.

### 3.5. Performance Discrimination and Assessment Evidence

Performance discrimination and other assessment-related evidence were analyzed separately from educational effectiveness. Associations between simulator performance and prior training or clinical experience were interpreted as assessment or relations-to-other-variables evidence and were not considered, by themselves, evidence of learning resulting from simulator-based training. Rogers-Vizena et al. evaluated unilateral cleft lip repair performance in 26 residents and fellows using blinded video assessment with the procedure-specific Unilateral Cleft Lip Repair (UCLR) instrument and a modified Objective Structured Assessment of Technical Skills (OSATS). The UCLR instrument comprised 18 items covering marking, procedural performance, and the final result. Total possible score ranges for UCLR and modified OSATS were not explicitly stated in the main article; detailed scoring instruments were referenced in its Supplementary Materials. Total UCLR score correlated significantly with training level (r = 0.4842, *p* = 0.0122), with significant associations also reported for procedure-specific performance and result subscores. For the total UCLR score, the interrater consistency ICC was 0.686 (95% CI, 0.413–0.846), whereas the agreement ICC was 0.618 (95% CI, 0.237–0.820). Corresponding modified OSATS estimates were 0.290 (95% CI, −0.103 to 0.604) and 0.217 (95% CI, −0.0956 to 0.522). These estimates indicate uncertainty in interrater reliability, particularly for modified OSATS [23]. In a subsequent study, training level was also associated with baseline UCLR (r = 0.550, *p* = 0.004) and OSATS scores (r = 0.454, *p* = 0.020) [35]. These findings provided relations-to-other-variables evidence that simulator-based performance reflected participants’ prior level of training. Kaimi et al. similarly demonstrated known-groups discrimination, with staff surgeons outperforming trainees during the first simulation session on both the cleft lip-specific assessment scale and the global rating scale (*p* < 0.05) [46]. The authors reported mean item-level ICCs of 0.72 for the cleft lip-specific scale and 0.70 for the global rating scale. Confidence intervals were reported for individual items rather than for these mean ICCs. For example, cleft lip-specific item ICCs included 0.648 (95% CI, 0.435–0.802) for muscle dissection and 0.816 (95% CI, 0.695–0.900) for mucosal dissection; global item ICCs included 0.595 (95% CI, 0.399–0.759) for knowledge of instruments and 0.789 (95% CI, 0.637–0.887) for knowledge of the procedure. Mean Cronbach α values were 0.90 and 0.88, respectively [46]. Saldanha et al. reported associations between participants’ prior experience and anthropometric outcomes [44]. These findings were retained as limited relations-to-other-variables evidence because they reflected cross-sectional associations with previous experience rather than change resulting from simulator-based training.

### 3.6. Cost and Implementation Characteristics

Implementation-related characteristics, including cost, manufacturing complexity, reusability, fabrication requirements, and availability, were inconsistently reported across the included publications. Importantly, reported monetary values referred to different cost components and were therefore not considered directly comparable estimates of total simulator cost, affordability, or cost-effectiveness.

Several publications provided specific manufacturing or per-use cost estimates. Chen et al. reported that the reusable polylactic acid mold used to produce the silicone simulator cost approximately USD 5, whereas each expendable silicone cleft lip model cost less than USD 2; a single reusable mold could be used to produce multiple silicone models [47]. Kantar et al. reported a production cost of USD 1.84 per unilateral cleft lip stone model, comprising approximately USD 0.64 for polyvinyl siloxane and USD 1.20 for SnapStone. Production required approximately 10 min per model, and the stone model could be reused when surgical markings were made in pencil and subsequently erased [36]. Reighard et al. reported manufacturing costs of USD 11.43 for the reusable molding system and USD 4.59 for each consumable cleft lip model [20]. In contrast, Podolsky et al. reported that disposable high-fidelity cleft lip simulator cartridges cost approximately USD 250 per use [15].

Stanek et al. directly compared reduced- and high-complexity simulators using estimates that incorporated both material costs and manufacturing labor, with labor valued at USD 75 per hour. The reduced-complexity simulator was estimated to cost approximately USD 89 per unit and contained no reusable components, whereas the high-complexity simulator required an approximately USD 345 single-use component in addition to an approximately USD 200 reusable base. The authors also reported a higher production-failure rate and an approximately fourfold greater labor requirement for the high-complexity simulator [50].

Other publications described implementation strategies without providing directly comparable monetary estimates. For example, the open-source simulator described by Teuber Lobos et al. used a reusable three-dimensionally printed mold and bony structure, with only the silicone soft-tissue component requiring replacement between training sessions [45]. Across the literature, equipment costs, manufacturing labor, maintenance, production failures, commercial availability, and lifecycle costs were not reported consistently. Consequently, the available evidence permits description of reported costs, fabrication requirements, and reusability, but does not support a standardized economic comparison or formal assessment of cost-effectiveness across simulator types.

### 3.7. Validity Evidence According to the Messick Framework

Following independent reassessment using the final operational criteria and consensus adjudication, Content-related validity evidence was not reported or not assessable in 11 of 24 publications (45.8%); 10 publications (41.7%) received a rating of 1 and three (12.5%) received a rating of 2. Supporting Content evidence was therefore identifiable in 13 publications (54.2%). Ratings of 1 reflected limited, indirect, or methodologically incomplete evidence that the content of a defined assessment score, instrument, or observation represented the intended construct or procedure, whereas ratings of 2 reflected strong, direct, and methodologically robust evidence, such as structured expert consensus, cognitive task analysis, or formal content evaluation. Strong Content evidence was identified in Reighard et al., Teuber Lobos et al., and Kaimi et al. [20,45,46].

Response Process was not assessable in five publications (20.8%); 14 publications (58.3%) received a rating of 1 and five (20.8%) received a rating of 2. Limited evidence most commonly reflected partial standardization of administration or scoring, whereas stronger evidence incorporated safeguards such as blinded independent rating, standardized video-based scoring, or formal rater calibration [23,35,37,46,51]. Supporting Response Process evidence was therefore identified in 19 of 24 publications (79.2%), making it the most frequently supported Messick domain.

Internal Structure was not assessable in 20 of 24 publications (83.3%); one publication (4.2%) received a rating of 1 and three (12.5%) received a rating of 2. The supporting publications reported formal interrater and/or intrarater reliability analyses, with internal-consistency analysis additionally reported in one publication [23,37,46,51].

Relations to Other Variables was not assessable in 18 publications (75.0%); one publication (4.2%) received a rating of 0, three (12.5%) received a rating of 1, and two (8.3%) received a rating of 2. Supporting evidence was therefore identified in five publications (20.8%). Evidence in this domain required an explicit relationship between simulator-derived outcomes and an external variable relevant to the intended interpretation; subjective confidence, realism, or perceived usefulness alone were insufficient. 

Consequences were not assessable in any of the 24 publications (100%) (Table 2 and Table 3). No publication reported a formally derived proficiency threshold, pass/fail standard, or progression criterion.

### 3.8. Inter-Rater Agreement Before Consensus

Both reviewers independently reassessed all 24 included publications using the final operational criteria. Inter-reviewer agreement was calculated from these updated independent ratings before consensus adjudication, separately for each Messick domain and for the adapted McGaghie classification. Across 144 classifications (24 publications × six classifications), the reviewers agreed on 126 ratings (87.5%); 18 disagreements (12.5%) required consensus adjudication. Disagreements occurred in Content (nine publications), Response Process (six publications), and Relations to Other Variables (three publications). Internal Structure, Consequences, and McGaghie classification showed complete raw agreement. Domain-specific agreement and Cohen’s κ values are presented in Table 4.

Agreement for the Content domain remained relatively low after independent reassessment using the final operational criteria (62.5%; κ = 0.379). This suggests continuing difficulty in distinguishing evidence supporting the content of a defined assessment from broader simulator characteristics, such as anatomical realism, procedural fidelity, or perceived educational value. Although consensus resolved the final classifications, uncertainty about the reproducibility of these judgments remains.

### 3.9. Educational Effectiveness According to the McGaghie Framework

Educational effectiveness was classified according to the highest level of educational outcome demonstrated in each publication. Publications that did not report learner-reaction or learning outcomes were not automatically assigned Level 1.

Two publications (8.3%)-the Rogers-Vizena simulator-development study and the Rogers-Vizena competency-assessment study did not evaluate educational effectiveness and were categorized as “educational effectiveness not evaluated” [21,23]. Fourteen publications (58.3%) were classified as Level 1 because their highest educational outcome consisted of learner reaction, perceived usefulness, satisfaction, realism, confidence, or self-reported knowledge or skill [15,17,19,20,24,36,38,39,42,43,44,45,49,50]. Eight publications (33.3%) reached Level 2 by demonstrating objectively measured improvement in knowledge or performance following an educational intervention or repeated simulation [35,37,40,41,46,47,48,51].

Objective differences associated only with participants’ previous experience or training level, as described in Section 3.5, were not considered sufficient for Level 2 classification. Level 2 required objectively measured improvement following training or repeated simulation. Accordingly, Kaimi et al. reached Level 2 on the basis of trainees’ objectively measured improvement across sequential simulation sessions, independently of the baseline performance-discrimination evidence reported in the same study [46].

No publication demonstrated Level 3 transfer to independently assessed clinical performance, Level 4 improvement in patient-level outcomes, or Level 5 organizational, healthcare-system, or population-level effects (Table 5).

### 3.10. Methodological Quality Assessment

All 24 included publications met both MMAT screening criteria, with clearly stated research questions and data adequate to address them. Methodological limitations varied according to study design. Among the four randomized controlled trials, baseline comparability and adherence to the assigned intervention were judged adequate in all studies. However, the adequacy of the randomization procedure was rated as “Can’t tell” in three of four studies, and outcome-assessor blinding was either not performed or insufficiently reported in two studies.

Among the nine quantitative non-randomized studies, administration of the intervention as intended was judged adequate in all studies, whereas completeness of outcome data was rated as “Yes” in six studies and as “Can’t tell” in three. The latter included the Rogers-Vizena et al. retention study, in which 21 of 26 participants completed the approximately 3-month follow-up and the characteristics of participants lost to follow-up and explicit handling of missing follow-up data were not reported. However, potential confounding was not accounted for in eight of nine studies, and representativeness of the target population was judged adequate in only one study. Measurement methods were rated as appropriate in five studies and as “Can’t tell” in four.

The nine quantitative descriptive studies showed consistently appropriate statistical analyses, whereas representativeness of the study samples was frequently unclear. Measurement methods were rated as appropriate in four studies, inappropriate in four, and as “Can’t tell” in one. The two mixed-methods studies combined quantitative pre/post survey outcomes with thematic analysis of narrative feedback, with meaningful integration occurring primarily at the interpretation stage, where quantitative changes in expertise, confidence, or attitudes were considered alongside qualitative findings concerning participants’ experiences, perceived educational value, and areas for course improvement. However, both showed limitations within their individual methodological components, particularly regarding participant representativeness, measurement reporting, and control of confounding. The complete MMAT assessment is presented in Supplementary File S7.

## 4. Discussion

Traditional surgical training has relied on apprenticeship-based learning, but contemporary education must increasingly balance technical independence with patient safety, working-hour restrictions, limited operative exposure, and competency-based progression [52,53]. Simulation offers a controlled setting for procedural rehearsal and structured feedback before clinical performance [54]. This is particularly relevant to cleft lip repair, which requires precise surgical planning and reconstruction and in which technical inaccuracies may contribute to asymmetry or the need for secondary correction [9,11]. Opportunities for repeated exposure to pediatric plastic surgery procedures may be limited [55], and several studies included in this review involved residents or fellows with little or no previous independent experience in cleft lip repair [45,46].

### 4.1. Educational Effectiveness and Clinical Transfer

This systematic review identified 24 publications evaluating simulation-based education, training, or assessment in cleft lip surgery. Fourteen publications (58.3%) were classified as McGaghie Level 1 [15,17,19,20,24,36,38,39,42,43,44,45,49,50], and eight (33.3%) as Level 2 [35,37,40,41,46,47,48,51], while none demonstrated Level 3 clinical transfer, Level 4 patient-level benefit, or Level 5 organizational effects. Two publications (8.3%) did not evaluate educational effectiveness and were therefore not assigned a McGaghie level.

Although confidence, perceived knowledge, procedural familiarity, satisfaction, and educational value frequently improved, these outcomes do not establish technical competence [17,24,35,36,44,49,50]. In contrast, nine publications reported objective educational or assessment outcomes, including measured improvement or performance discrimination [23,35,37,40,41,46,47,48,51]. Wang et al. and Chen et al. reported higher post-intervention knowledge or operative scores following simulator-assisted teaching, while Plana et al. demonstrated greater improvement in surgical marking with digital simulation than with textbook-based learning [41,47,51]. These studies provide direct comparative evidence of educational benefit, although the methodological limitations identified by MMAT warrant cautious interpretation. Kantar et al. reported objective improvement within the digital-simulation group; however, an adequate direct between-group comparison of change was not reported. The findings therefore support improvement following the intervention but do not establish superiority over textbook-based learning [37].

Evidence from repeated-training studies suggests that educational gains may extend beyond a single simulator exposure. In the study by Rogers-Vizena et al., improvements in procedure-specific performance, OSATS scores, confidence, and self-assessed knowledge remained detectable at approximately three months; however, only 21 of 26 participants completed follow-up, and the characteristics of those lost to follow-up and the handling of missing follow-up data were not reported. The retention findings should therefore be interpreted cautiously. Kaimi et al. observed progressive improvement across sequential simulated repairs. Overall, these findings suggest a potential role for repeated simulation in skill development and short-term retention, although the optimal number and spacing of training sessions remain unclear [35,46].

Despite these educational benefits, clinical transfer was not demonstrated in the included studies. The included simulators addressed different components of cleft lip repair, ranging from anatomical orientation, surgical marking, and incision planning to tissue dissection, orbicularis oris reconstruction, multilayer closure, and nasal reconstruction. The extent to which these procedural components were reproduced varied across models [15,21,36,39,40,51]. No included study demonstrated improved performance during actual cleft lip surgery or improvement in patient-level outcomes (Table 5). Accordingly, self-reported preparedness, confidence, or perceived changes in clinical practice were not considered evidence of Level 3 clinical transfer [33].

The strength of evidence supporting the educational findings varied according to study design, outcome type, and methodological quality. Direct comparative evidence favoring simulator-assisted education was provided by the randomized studies by Wang, Chen, and Plana, which assessed objective knowledge or simulated technical performance [41,47,51]. Nevertheless, these findings should be interpreted with some caution, as randomization procedures were insufficiently described in three of the four randomized trials, outcome-assessor blinding was absent or unclear in two, and completeness of outcome data was uncertain in one. Evidence from non-randomized and uncontrolled studies was less conclusive, particularly because potential confounding was frequently not adequately addressed. Similarly, findings based primarily on self-reported confidence, perceived knowledge, satisfaction, or educational value provide useful information on learner experience and perceived benefit but offer more limited evidence of educational effectiveness [17,21,24,36,44,49,50]. Consequently, conclusions regarding objective skill acquisition were based primarily on comparative studies with objective outcome measures, whereas uncontrolled, cross-sectional, and predominantly self-reported findings were interpreted more cautiously as evidence of feasibility, acceptability, or perceived educational benefit.

### 4.2. Validity Evidence and Competency Assessment

Evaluation using the Messick framework revealed substantial variation in the strength of validity evidence across domains. Response Process was the most frequently supported domain, with evidence identified in 19 of 24 publications (79.2%), whereas Content evidence was identified in 13 of 24 publications (54.2%). Strong Content evidence was limited to three studies and reflected structured approaches such as cognitive task analysis, formal content evaluation, or expert consensus [20,45,46].

However, anatomical realism and procedural fidelity should not be regarded as synonymous with validity. A simulator may reproduce cleft anatomy or operative steps accurately while still lacking evidence that observations or scores derived from its use are reliable and appropriate for assessment [31].

Evidence for Response Process was variable, while evidence for Internal Structure and Relations to Other Variables was considerably less frequent. Stronger studies incorporated standardized administration, blinded or independent rating, rater calibration, reliability analyses, or associations between simulator performance and external indicators of competence such as training level. Together with improvements observed across repeated simulated repairs, these findings suggest that selected simulator-based assessments may have value for monitoring technical performance within the simulated environment [23,35,37,46,51].

No study provided assessable Consequences evidence, and none established a formally derived proficiency threshold, pass/fail standard, or progression criterion. Thus, although some tools show potential for performance assessment and tracking progress, current evidence remains insufficient to support simulator performance as an independent determinant of readiness for clinical practice.

Inter-reviewer disagreement was greatest for Content and Response Process, whereas agreement was complete for Internal Structure and the McGaghie classification (Table 4). Content agreement remained relatively low under the final operational criteria (62.5%; κ = 0.379), suggesting that the distinction between broader simulator characteristics and evidence supporting a defined assessment interpretation remained difficult to apply consistently [31]. Although consensus resolved the final classifications, uncertainty about the reproducibility of the independent judgments remains.

### 4.3. Simulator Fidelity, Cost, and Accessibility

Simulator complexity, cost, accessibility, and educational value varied substantially across the included studies. High-fidelity models could reproduce multiple tissue layers, muscle repair, multilayer closure, and components of primary rhinoplasty, but often required specialized fabrication methods and were among the more costly approaches [15,20,39,50]. Reported costs ranged from less than US$2 for individual silicone models to several hundred US dollars for high-complexity simulator components [47,50].

In the available comparative evidence, greater simulator complexity did not demonstrate superior educational outcomes. Stanek et al. found no statistically significant difference in self-assessed improvement or confidence between reduced- and high-complexity simulators despite markedly different costs: approximately US$89 for the reduced-complexity model versus approximately US$345 for a disposable high-complexity component plus an approximately US$200 reusable base. Although these subjective outcomes cannot establish equivalent objective skill acquisition, they indicate that greater physical complexity and higher cost should not be assumed to provide greater educational value [50].

Implementation-related characteristics were inconsistently reported. Information on commercial availability, manufacturing requirements, durability, reusability, and recurring costs was often incomplete, while several models required three-dimensional printing, silicone casting, patient-derived manufacturing processes, or other specialized methods. Several simulators were developed within individual academic centers and evaluated primarily as locally produced training models or prototypes, which may further limit straightforward implementation in other settings. This suggests that practical accessibility remains insufficiently characterized alongside simulator realism and educational value [15,20,21,39,45,47].

### 4.4. Comparison with Simulation in Other Areas of Plastic and Cleft Surgery

The findings of this review are broadly consistent with the wider plastic surgery simulation literature. Foppiani et al., in a systematic review and meta-analysis of 18 studies, reported significant improvements in objective technical performance measured using OSATS and in self-reported confidence following simulation-based training [56]. Similarly, the present review identified objective improvements in knowledge, surgical marking, and simulated technical performance in a subset of studies.

Limited translation of educational gains into clinical practice also appears to be a broader challenge in plastic surgery simulation rather than one specific to cleft lip training. Lastoria et al. reviewed 116 studies across multiple areas of plastic surgery using the Messick validity framework and McGaghie translational outcomes hierarchy. Only one model achieved an effectiveness level above Level 3, and no model received a rating above 2 across all Messick validity domains [12]. The predominance of Level 1–2 evidence in the present cleft lip-specific review is therefore consistent with the generally low translational effectiveness reported across the wider field.

Similar limitations were identified in earlier cleft surgery literature. Kantar et al. described 16 cleft surgery simulators across 22 studies and highlighted substantial variation in fidelity, purpose, cost, availability, and supporting evidence. The authors emphasized the need for stronger evidence of simulator efficacy and standardized cleft-specific assessment tools [26]. The present review indicates that, although objective educational outcomes have now been demonstrated in a subset of studies, limitations in validation and clinical transfer remain.

### 4.5. Implications for Training and Future Simulator Development

The findings suggest that simulator complexity should be matched to the intended educational task rather than maximized as an objective in itself. Simpler models may be sufficient for anatomical orientation, cleft marking, incision planning, and early procedural familiarization, whereas more detailed multilayer models can support tissue dissection, orbicularis reconstruction, layered closure, and complete procedural rehearsal [15,21,36,39,40,51]. Future simulator development should therefore balance the level of fidelity required for the intended training objective with affordability, reproducibility, and practical accessibility [45,47,50].

Simulation may be particularly valuable in residency and fellowship training, where opportunities for repeated operative exposure to cleft lip repair can be limited. In the study by Kaimi et al., none of the participating residents had previously performed an independent cleft lip repair, and only one fellow had independently performed two procedures. Repeated simulator use in the same study resulted in progressive improvement in technical performance [46]. More broadly, limited operative exposure to core pediatric plastic surgery procedures has been documented across Canadian plastic surgery residency programs by D’Abbondanza et al. [55]. Challenges related to limited training opportunities in craniofacial and cleft surgery have also been highlighted by Teuber Lobos et al., who developed a low-cost, open-source simulator specifically to facilitate regular teaching and training of cleft anatomy and surgical techniques [45]. These findings support the role of reproducible simulation models as a means of providing repeated exposure to key operative steps that may be difficult to obtain consistently through clinical training alone.

Where simulators are intended not only for training but also for assessment, their development should be accompanied by structured evaluation using objective performance measures, reliability testing, and assessment of relationships with experience level or changes in performance over time. Realism alone does not establish that a simulator can reliably measure technical competence [23,31,37,46,51]. Demonstrating that simulator-based performance predicts independently assessed performance during actual cleft lip surgery would provide stronger evidence for incorporating simulator-based performance criteria into cleft surgery training.

### 4.6. Limitations

Several limitations should be considered. The included studies were highly heterogeneous in simulator modality, cleft phenotype, participant experience, training format, assessment methods, outcomes, and follow-up, precluding meta-analysis and necessitating narrative synthesis. Sample sizes were also frequently small, particularly in simulator-development and expert-evaluation studies, limiting the precision and generalizability of the findings.

A substantial proportion of studies relied on subjective endpoints such as confidence, realism, satisfaction, and perceived educational value. These outcomes were considered measures of feasibility and acceptability but not evidence of technical competence or clinical transfer. Although eight publications demonstrated objectively measured improvement in knowledge or performance following an educational intervention or repeated simulation, none evaluated transfer to independently assessed clinical performance or patient-level outcomes (Table 5). Where simulator developers were also involved in evaluating their models, subjective assessments of realism, usability, or educational value may have been susceptible to favorable assessment bias. In addition, the predominance of studies reporting favorable intervention outcomes raises the possibility of publication or reporting bias, which may have led to an overrepresentation of positive findings in the available evidence base.

No formal assessment of reporting or publication bias was performed, and the certainty of evidence was not evaluated using a formal certainty-of-evidence framework. Therefore, preferential publication or reporting of positive educational findings cannot be excluded and may have contributed to an overestimation of the apparent educational benefits of simulation. The MMAT 2018 assessment identified recurring methodological limitations across study designs. Randomization procedures and outcome-assessor blinding were frequently unclear in randomized studies, while most non-randomized studies did not adequately account for potential confounding. Concerns regarding participant representativeness and measurement quality were also common across non-randomized, descriptive, and mixed-methods studies (Supplementary File S7).

Application of the Messick framework was limited by inconsistent terminology and reporting of validity evidence across studies. Ratings were therefore based only on explicitly reported evidence and predefined domain-specific criteria. In particular, evidence relating to reliability, relationships with external indicators of competence, and consequences of simulator-based assessment was infrequently reported (Table 2 and Table 3). Similar challenges in interpreting validity evidence have been reported in the simulation-based assessment literature [31].

The literature search was conducted in four bibliographic databases, and eligibility was restricted to English-language full-text articles published between 2016 and 2026; therefore, relevant studies not indexed in these databases or published outside these limits may have been missed. Additionally, backward and forward citation searching was not performed, which may have resulted in potentially eligible studies not identified through the database searches being missed. Although the review protocol was developed a priori, PROSPERO registration was completed after title and abstract screening had been completed, and the Messick and adapted McGaghie frameworks were introduced subsequently as methodological refinements. Consequently, the publicly registered record cannot independently establish prespecification of all methodological decisions made during the review process.

Implementation-related variables, including cost, commercial availability, manufacturing complexity, durability, and reusability, were inconsistently reported. Consequently, conclusions regarding accessibility and affordability should be interpreted as reflecting the available published evidence rather than demonstrating the absence of commercially available or readily implementable cleft lip simulators.

Finally, this review was restricted to cleft lip simulation. Studies focused exclusively on cleft palate or broader craniofacial procedures without separately extractable cleft lip outcomes were excluded. This increased procedural specificity but limited direct comparison with the broader cleft surgery literature. Despite these limitations, the review provides a procedure-specific synthesis integrating educational effectiveness, validity evidence, methodological quality, and implementation characteristics of cleft lip simulation.

## 5. Conclusions

Simulation-based training in cleft lip surgery can provide measurable educational benefits beyond learner satisfaction and self-reported confidence, including improvements in knowledge, surgical marking, and simulated technical performance. Repeated simulation may also support skill development and short-term retention.

Confidence in these findings is limited by small samples, incomplete reporting of randomization and assessor blinding, potential confounding, and incomplete follow-up.

However, the evidence included in this review remains limited to educational and simulated settings, with no demonstrated transfer to independently assessed clinical performance or patient-level outcomes.

Available evidence does not support the assumption that greater simulator complexity and cost necessarily provide greater educational value. However, the absence of a statistically significant difference in subjective outcomes between simulator types does not establish equivalent objective skill acquisition. A task-specific approach that balances fidelity with accessibility, affordability, and reproducibility may therefore be preferable. Future development should combine simulator innovation with rigorous assessment of validity, reliability, and educational effectiveness, while determining whether simulator-based performance translates into improved performance during actual cleft lip surgery.

## Figures and Tables

**Table 1:** Characteristics of the 24 included publications.

Publication	Study Design	Participants/Experience	Simulator/Modality	Training or Assessment Format	Comparator	Main Outcomes	Follow-Up	Key Finding/Interpretation
Zaga-Galante et al. [39]	Simulator development and expert evaluation	*n* = 11 experienced cleft surgeons; 82% had >10 years of cleft-surgery experience; 63.6% had performed >80 bilateral repairs	Simulare Medical high-fidelity bilateral cleft lip simulator; physical	Hands-on bilateral cleft lip repair followed by a 5-point post-use questionnaire	None	Anatomical accuracy, realism, simulator features, perceived training value	Immediate post-simulation only	All participants completed the repair; mean realism/anatomical accuracy 3.7/5 and training value 4.6/5. Expert-perception evidence; no objective learning outcome.
Lutz et al.—bilateral [19]	Simulator development/proof-of-concept with single-group educational evaluation	*n* = 12 trainees/clinicians; low practical cleft experience; none had independently performed bilateral cleft lip repair	Ex vivo porcine snout-disc bilateral cleft lip model; biological physical model	Lecture on model construction and Millard repair, procedural demonstration, then hands-on repair	None	Self-perceived theoretical knowledge, practical skills, comprehension, educational value	Immediate post-training; no longer-term follow-up	High self-reported gains after a single course; no objective performance measure or control group.
Lutz et al.—unilateral [38]	Prospective single-group educational intervention	*n* = 16 beginners: 9 CMF residents, 3 oral-surgery residents, 4 licensed medical/dental professionals; limited/no prior cleft experience	Ex vivo porcine snout-disc unilateral cleft lip model; biological physical model	Instructor-led workshop followed by independent unilateral repair	None	Self-perceived comprehension, theoretical knowledge, practical skills, educational value	Baseline experience assessed before training; educational perceptions assessed immediately post-training; no longitudinal follow-up.	Participants reported improved understanding, knowledge and practical skills; no objective performance assessment or control group.
Vogl et al. [40]	Prospective educational evaluation with subjective and objective outcomes	*n* = 38: 10 OMFS surgeons and 28 dental students; specialists had variable experience, students essentially none	Unilateral and bilateral ex vivo porcine cleft lip models; high-fidelity biological models	Instructor-led theoretical teaching plus hands-on unilateral and bilateral repair	No concurrent non-simulation control; specialist, online-student and face-to-face student groups	Realism, perceived educational value, task performance and objective 3D-scan outcomes	Immediate; student tasks assessed pre/post	Mean task scores increased from 4.44 to 6.07 for unilateral cleft landmarks and from 2.31 to 3.44 for bilateral landmark matching; each task had a maximum of 7 points. These represent increases of 1.63 and 1.13 points. No concurrent non-simulation control or confidence intervals for these changes were reported.
Wang et al. [41]	Randomized comparative educational trial	*n* = 40 senior stomatology students; learner population without reported clinical cleft-repair experience	Commercial 3D-printed high-fidelity cleft lip simulator; physical/haptic	PBL teaching with case analysis, discussion, instruction, demonstration and hands-on simulation	3D-simulator-assisted PBL (*n* = 20) vs. conventional PBL using a low-density sponge model (*n* = 20)	Theoretical knowledge, operative skills, specific surgical-task scores, learner-reported educational outcomes	Pre-training baseline and post-training assessment; no longitudinal follow-up reported	Post-training theoretical scores were higher with 3D-simulator-assisted PBL: median 80 (IQR 70–87.5) versus 70 (IQR 42.5–80) points on a 100-point test (*p* = 0.029). All six operative domains favored the 3D-PBL group (*p* = 0.001 to 0.005, or *p* < 0.001). Confidence intervals for the between-group effects were not reported. Findings concern the educational packages compared.
Riedle et al. [42]	Simulator development and single-group evaluation	*n* = 20 surgical residents; mean PGY 2.5 ± 1.6; mean 5.8 ± 8.0 assisted cleft lip/palate repairs	Generic 3D-printed silicone unilateral cleft lip/palate model; physical/haptic	Instructor-led hands-on simulation with pre/post questionnaire	None	Realism, usability, cutting/suturing characteristics, completion of repair, perceived educational value	Before and immediately after simulation	83% completed the simulated repair and all reported perceived improvement; no objectively measured performance gain.
Kantar et al.—5-year digital assessment [43]	Observational descriptive study of use and global reach	Survey: 132 registered users and 42 Smile Train partners; platform analytics included 2865 new users and 4086 active users	CleftSim internet-based digital simulator; interactive 3D/video platform	Self-directed web-based learning	None	Usefulness, use for teaching, demographics, usage patterns and global reach	Post-use survey; platform analytics through June 2017	Broad global reach (136 countries); 88% of surveyed users found the simulator useful. Descriptive learner-reaction/use data, not objective learning.
Rogers-Vizena et al.—simulator development [21]	Pilot simulator-development and preliminary validation study	*n* = 3 cleft surgeons; specific prior case volume not reported	High-fidelity unilateral cleft lip simulator; physical	Individual simulated repair with pre/post 3D surface scanning	Simulator-derived nasolabial changes compared with patient-derived operative change patterns	Anatomical/haptic realism, feasibility of key repair steps, similarity of simulator vs. patient nasolabial changes	During single simulator-use episode; no educational follow-up	Key operative steps were feasible; labial changes resembled patient patterns more closely than nasal changes. Simulator-development/assessment evidence; educational effectiveness not evaluated.
Reighard et al. [20]	Simulator development and expert evaluation	*n* = 5 cleft surgeons from 3 institutions; mean 6.4 ± 3.5 years in practice; 40–150 prior repairs	Novel low-cost 3D-printed/silicone composite cleft lip repair simulator; physical	Independent hands-on repair with standard instruments followed by structured expert evaluation	None	Physical attributes, realism, value/relevance and ability to perform key repair tasks	After simulator repair; no longitudinal follow-up reported	Expert ratings were generally sufficient-to-excellent and suggested only minor refinement before training use; no learner performance change was tested.
Khoo et al. [17]	Prospective single-group pre/post educational evaluation	*n* = 11 plastic-surgery trainees (SET 1–5); mixed prior cleft exposure, including two with prior primary-surgeon experience	Simulare Medical unilateral cleft lip simulator; high-fidelity physical model	Video demonstration followed by instructor-led hands-on repair	None	Self-reported confidence, knowledge, technical skill and anxiety	Immediate pre/post	Mean total procedural-confidence score increased by 53.5%; educational effect was learner-reported rather than objectively measured.
Podolsky et al. [15]	Pilot expert evaluation	*n* = 4 expert cleft surgeons; each had performed >100 cleft lip repairs	High-fidelity bench-top cleft lip simulator (Simulare Medical); physical	Complete simulated cleft lip repair including primary rhinoplasty using real instruments	None	Realism, anatomical accuracy and perceived training value	Assessment timing not explicitly reported; no longitudinal follow-up reported	All seven simulated repairs were completed and experts considered the model realistic and valuable; qualitative expert evaluation, not a learning-effect study.
Saldanha et al. [44]	Prospective single-arm pre/post curriculum study	*n* = 16 residents/fellows; mostly minimal prior cleft exposure and simulation-naive	Proprietary high-fidelity cleft lip simulator; physical bench-top model	Small-group bootcamp: lecture, instructional video, hands-on simulation and Q&A	None	Self-assessed knowledge/skills, procedural confidence and 3D lip/nasal symmetry	Pre-session, post-lecture and post-simulation; no long-term follow-up	Self-assessed knowledge/skills improved after the multimodal curriculum. Objective anthropometric findings were cross-sectional associations with experience, not objective pre/post learning attributable to simulation.
Teuber Lobos et al. [45]	Simulator development with expert/trainee evaluation	*n* = 16: 10 trainees (residents/undergraduates) and 6 experienced OMFS surgeons	Open-source hybrid cleft lip/palate simulator; 3D printing + silicone casting	Guided hands-on unilateral cleft lip/palate repair followed by standardized questionnaire	No control; trainee and expert evaluator groups	Anatomical appearance, tissue/haptic realism, procedural realism and perceived educational utility	Immediate post-simulation	Most participants rated anatomy, tissue behavior and teaching utility favorably; implementation is open-source, but educational evidence is primarily subjective.
Rogers-Vizena et al.—retention [35]	Prospective single-blinded repeated-measures interventional study	*n* = 26 residents/fellows (PGY3-fellow); median prior cleft repairs 5.5 (range 0–45); 21 returned at follow-up	High-fidelity unilateral cleft lip simulator; physical/haptic	Simulated repair with structured debriefing; simulations 1 and 2 back-to-back, simulation 3 at ~3 months	Within-participant repeated measures	Modified OSATS, UCLR, self-assessed knowledge/confidence and procedural confidence	Immediate repeated assessment and ~3-month follow-up (21/26)	Adjusted mean ± SE OSATS scores were 16.095 ± 0.593, 17.476 ± 0.466, and 16.905 ± 0.441 across simulations 1–3; corresponding UCLR scores were 43.714 ± 1.537, 48.190 ± 0.950, and 47.667 ± 1.024. Both objective measures remained significantly above baseline at approximately 3 months. Only 21/26 participants returned; attrition and the absence of a concurrent control limit interpretation.
Rogers-Vizena et al.—competency [23]	Prospective competency-assessment/construct-validity cohort study	*n* = 26 residents/fellows (PGY3-fellow); prior repairs 0–45, median 5.5	High-fidelity unilateral cleft lip simulator; physical	Single independent simulated repair with blinded video scoring	None; training level and cumulative prior experience were examined as external variables	UCLR, modified OSATS and 3D surface symmetry	Single assessment	UCLR performance correlated with training level; this is performance-discrimination/validity evidence, not evidence that simulator training caused learning. Educational effectiveness not evaluated.
Kaimi et al. [46]	Simulator evaluation with repeated-simulation learning-curve and known-groups assessment	*n* = 13: 4 residents, 6 pediatric-plastic-surgery fellows and 3 expert staff surgeons; residents had no independent prior repairs	Simulare Medical high-fidelity unilateral cleft lip simulator; physical	2 h didactic session; trainees completed 3 simulated repairs at 1–4 week intervals with feedback after each session; staff surgeons completed 1 benchmark session; blinded video rating	Staff surgeons as expert benchmark; within-trainee repeated sessions	Cleft lip-specific technical score, global rating scale, interrater reliability and internal consistency	Three trainee simulation sessions at 1–4 week intervals; no additional longitudinal follow-up reported	Across three trainee sessions, mean ± SD cleft lip-specific scores increased from 28.5 ± 5.85 to 36.3 ± 9.12 and 43.5 ± 9.02 (*p* = 0.015); global scores increased from 12.9 ± 3.77 to 17.3 ± 3.38 and 21.1 ± 4.73 (*p* = 0.027). Staff outperformed trainees at session 1. Learning-curve findings lacked a concurrent training control; the source reports an inconsistent global score maximum.
Chen et al. [47]	Simulator development plus randomized comparative educational study	*n* = 53 undergraduate dental students; no prior cleft-repair classroom or practical training	Patient-derived personalized silicone cleft lip model produced with CAD/CAM; physical	Instructor-led class; experimental group received physical-model demonstration and examination	Model + PowerPoint (experimental *n* = 27) vs. PowerPoint alone (control *n* = 26)	10-item theoretical knowledge test and post-class self-assessment	Knowledge assessed pre/post; self-assessment post-class	Post-class total knowledge score favored simulator-assisted teaching: median 9 (IQR 8–9) vs. 6 (IQR 6–7), *p* < 0.001; selected anatomy/confidence items also favored the experimental group. Direct comparative knowledge evidence.
Kantar et al.—stone models [36]	Prospective within-subject comparative educational study	*n* = 13 plastic-surgery residents	Reusable 3D stone unilateral cleft lip marking model; physical	15 min self-study followed by cleft-lip markings on the stone model and a standardized photograph	3D stone model vs. 2D patient photograph within the same participants	Learner satisfaction: stimulation, interest, learning, clarity, perceived effectiveness and recommendation	Post-task survey; no longitudinal follow-up reported	Stone models were rated significantly more favorably across all subjective domains; objective skill acquisition was not measured.
Volk et al. [48]	Prospective uncontrolled pre/post educational study	*n* = 16: 15 plastic-surgery residents and 1 craniofacial fellow; junior and senior subgroups	Smile Train Virtual Surgery Simulator; web-based digital	Self-directed completion of anatomy, markings and procedure modules within 2 h	None	Objective knowledge and self-reported confidence	Immediately before and after 2 h session	Knowledge scores increased from 33.1% to 64.4% among junior residents and from 46.9% to 70.8% among senior trainees, corresponding to increases of 31.3 and 23.9 percentage points. No concurrent control was used; practice effects, repeated testing, and other educational exposure cannot be excluded.
Michaels et al. [49]	Prospective single-group pre/post course evaluation	*n* = 35 pediatric-otolaryngology fellows; graduates of 27 different residency programs; prior-year procedural experience varied by task (0–15 procedures)	3D-printed procedure simulators used in virtual telesimulation; cleft lip plus other pediatric procedures	Pre-course expert videos/PDFs followed by live teleconference with four procedural breakout stations	None	Self-reported procedural expertise, confidence, course attitudes and qualitative feedback	Pre-course; post-station; end-of-course	Self-reported expertise and confidence improved; supports feasibility/acceptability of telesimulation, but objective technical performance was not measured.
Stanek et al. [50]	Hybrid retrospective-prospective comparative cohort study	*n* = 52: plastic-surgery residents, OMFS residents, and craniofacial/pediatric plastic-surgery fellows; 26/group; median prior cleft-lip repairs 2.5 (HCS) vs. 2.0 (LCS)	Reduced-complexity vs. high-complexity physical cleft lip simulators	Bootcamp lecture followed by guided hands-on simulation in small groups; operative video available	Low-complexity simulator (*n* = 26) vs. high-complexity simulator (*n* = 26)	Self-assessed knowledge/skill, procedural confidence and implementation cost	Post-lecture/pre-simulation and immediate post-simulation	Mean ± SD self-assessed improvement was 3.7 ± 2.2 with the reduced-complexity simulator versus 2.7 ± 1.9 with the high-complexity simulator (*p* = 0.09); adjusted RR was 1.30 (95% CI, 0.96–1.77). Production-cost estimates favored the reduced-complexity model. Subjective outcomes and a nonsignificant difference do not establish equivalent objective skill acquisition or comparative cost-effectiveness.
Plana et al. [51]	Prospective randomized blinded comparative trial	*n* = 35 first-year medical students with no prior cleft or operating-room exposure	Smile Train Virtual Surgical Simulator; digital/web-based	20 min digital-simulation or textbook study, with blinded pre/post cleft-lip marking assessment; crossover for satisfaction survey	Digital simulation (*n* = 18) vs. textbook (*n* = 17)	Objective cleft-lip marking performance and learner satisfaction	Immediate pre/post; no longer-term follow-up	Greater improvement in marking performance with digital simulation than with textbook learning (between-group *p* < 0.001). Reported mean changes differ between the source abstract (5.81 ± 2.01 vs. 2.68 ± 1.49 points) and main Results (5.17 ± 1.86 vs. 2.11 ± 1.27 points). Neither set was treated as definitively correct.
Kantar et al.—digital simulation [37]	Prospective randomized blinded comparative trial	*n* = 13 plastic-surgery residents (PGY1–4), before PGY5 craniofacial rotation	Smile Train digital cleft simulator; 3D stone marking model and high-fidelity haptic model used for assessment	Standardized digital or textbook exposure followed by objective marking and simulated surgical-performance testing	Digital simulation (*n* = 6) vs. textbook (*n* = 7)	Surgical knowledge, confidence, marking performance, modified OSATS surgical performance and satisfaction	Immediate pre/post; satisfaction after crossover exposure	Within the digital-simulation group, knowledge increased from 33.5 ± 3.7 to 40.3 ± 4.4 (maximum 45; *p* = 0.03), marking performance from 2.9 ± 3.1 to 8.0 ± 2.5 (maximum 10; *p* = 0.03), and surgical performance from 8.2 ± 2.3 to 12.3 ± 2.5 (maximum 20; *p* = 0.04); values are mean ± SD. An adequate direct between-group comparison of change was not reported, so superiority over textbook learning was not established.
Chang et al. [24]	Multicenter quasi-experimental pre/post simulation-course study	*n* = 26: 9 pediatric-otolaryngology fellows and 17 senior residents (16 PGY5, 1 PGY3); 88% reported 0 cleft-lip procedures in the prior year	3D-printed high-fidelity unilateral cleft lip simulator within a broader pediatric-otolaryngology course; physical	Preparatory electronic materials followed by 45 min hands-on stations with two faculty per station	None	Self-rated expertise, confidence, attitudes toward simulation/3D printing and qualitative trainee feedback	Presurvey before the course; postsurveys at the conclusion of each simulation/course experience	Self-rated expertise and confidence increased, with cleft lip showing the largest effect sizes; no objective technical-performance assessment.

Note. 3D, three-dimensional; CI, confidence interval; CMF, craniomaxillofacial; IQR, interquartile range; OMFS, oral and maxillofacial surgery; OSATS, Objective Structured Assessment of Technical Skills; PBL, problem-based learning; PGY, postgraduate year; RR, relative risk; SD, standard deviation; SE, standard error; SET, Surgical Education and Training; UCLR, Unilateral Cleft Lip Repair. Values are reported as mean ± SD, adjusted mean ± SE, or median (IQR), as indicated. SD and IQR describe variability, whereas SE describes the precision of an estimated mean; neither represents a confidence interval for an intervention effect. Follow-up refers to the timing of outcome assessment reported in each publication. Where simulation was part of a multimodal curriculum, effects are attributed to the educational package as delivered rather than to the simulator alone. Rogers-Vizena competency [23] and retention [35] represent overlapping publications from the same underlying trainee cohort. Potential participant overlap between Kantar [36,37] and between the unilateral and bilateral Lutz publications [19,38] could not be excluded from the published reports. Chen et al. [47] report 53 randomized students in the Methods (27 experimental, 26 control), although the article’s results tables display *n* = 27 for each group; this table follows the Methods and reported total sample. Plana et al. [51] contain discrepant numerical change estimates between the abstract and main-text Results; both sets were retained and identified by source location, and neither was selected as definitively correct.

**Table 2:** Messick validity evidence and McGaghie educational effectiveness.

ID	Publication	Messick Validity Evidence					McGaghie Educational Effectiveness
		Content	Response Process	Internal Structure	Relations to Other Variables	Consequences	Level
1	Zaga-Galante et al. [39]	1	1	N	N	N	1
2	Lutz et al.—bilateral [19]	N	1	N	N	N	1
3	Lutz et al.—unilateral [38]	N	1	N	N	N	1
4	Vogl et al. [40]	1	1	N	0	N	2
5	Wang et al. [41]	1	1	N	N	N	2
6	Riedle et al. [42]	N	1	N	N	N	1
7	Kantar et al.—5-year digital assessment [43]	N	N	N	N	N	1
8	Rogers-Vizena et al.—simulator development [21]	1	1	N	1	N	Not evaluated
9	Reighard et al. [20]	2	1	N	N	N	1
10	Khoo et al. [17]	N	1	N	N	N	1
11	Podolsky et al. [15]	1	N	N	N	N	1
12	Saldanha et al. [44]	1	1	N	1	N	1
13	Teuber Lobos et al. [45]	2	1	N	N	N	1
14	Rogers-Vizena et al.—retention [35]	1	2	N	1	N	2
15	Rogers-Vizena et al.—competency [23]	1	2	1	2	N	Not evaluated
16	Kaimi et al. [46]	2	2	2	2	N	2
17	Chen et al. [47]	N	N	N	N	N	2
18	Kantar et al.—stone models [36]	N	N	N	N	N	1
19	Volk et al. [48]	N	N	N	N	N	2
20	Michaels et al. [49]	N	1	N	N	N	1
21	Stanek et al. [50]	N	1	N	N	N	1
22	Plana et al. [51]	1	2	2	N	N	2
23	Kantar et al.—digital simulation [37]	1	2	2	N	N	2
24	Chang et al. [24]	N	1	N	N	N	1

Note. Messick validity evidence was coded independently for each domain as follows: N = relevant evidence was not reported or could not be assessed; 0 = the domain was assessed, but the findings did not support the intended interpretation; 1 = limited, indirect, partial, or methodologically incomplete supporting evidence; and 2 = strong, direct, and methodologically robust supporting evidence. Domain ratings refer specifically to evidence supporting the interpretation of simulator- or assessment-derived observations or scores and should not be interpreted as measures of overall study quality. Ratings were considered independently and were not combined into a composite validity score. Educational effectiveness was classified separately using the adapted McGaghie framework. Not evaluated indicates that the publication did not assess learner reaction or learning outcomes and is not a McGaghie level. Level 1 denotes learner reaction or other subjective outcomes, whereas Level 2 denotes objectively demonstrated improvement in knowledge or technical performance following an educational intervention or repeated simulation, including retention of objectively measured gains within the simulated or educational setting. Performance discrimination based solely on prior training or experience was treated as assessment/validity evidence and was not considered sufficient for Level 2 classification.

**Table 3:** Summary of final consensus Messick validity evidence.

Domain	N, *n* (%)	0, *n* (%)	1, *n* (%)	2, *n* (%)
Content	11 (45.8%)	0 (0.0%)	10 (41.7%)	3 (12.5%)
Response process	5 (20.8%)	0 (0.0%)	14 (58.3%)	5 (20.8%)
Internal structure	20 (83.3%)	0 (0.0%)	1 (4.2%)	3 (12.5%)
Relations to other variables	18 (75.0%)	1 (4.2%)	3 (12.5%)	2 (8.3%)
Consequences	24 (100.0%)	0 (0.0%)	0 (0.0%)	0 (0.0%)

**Table 4:** Inter-rater agreement before consensus.

Domain/Classification	Exact Agreement	Disagreements	Cohen’s κ	Interpretation
Messick—Content	15/24 (62.5%)	9	0.379	Fair
Messick—Response Process	18/24 (75.0%)	6	0.617	Substantial
Messick—Internal Structure	24/24 (100%)	0	1.000	Perfect
Messick—Relations to Other Variables	21/24 (87.5%)	3	0.717	Substantial
Messick—Consequences	24/24 (100%)	0	Not estimable	Perfect raw agreement
McGaghie classification	24/24 (100%)	0	1.000	Perfect

Note. N, 0, 1, and 2 were treated as distinct categories. Cohen’s κ for Consequences is not estimable because both reviewers assigned the same single category (N) to all studies, resulting in zero between-category variance despite 100% raw agreement. Interpretation of Cohen’s κ followed the thresholds reported by Belur et al. [30].

**Table 5:** Adapted McGaghie translational outcome levels.

Level	Definition	*n*	%
Educational effectiveness not evaluated	No learner reaction or learning outcome	2	8.3%
1	Learner reaction, satisfaction, perceived usefulness, or self-reported confidence	14	58.3%
2	Objectively demonstrated improvement in knowledge or technical performance following an educational intervention or repeated simulation, including retention of objectively measured gains within an educational or simulated setting.	8	33.3%
3	Transfer of acquired skills to actual clinical practice	0	0.0%
4	Improvement in patient-level outcomes	0	0.0%
5	Organizational, healthcare-system, or population-level effects	0	0.0%

## Data Availability

The data generated and analyzed during this study are provided in the article and its Supplementary Materials.

## Supplementary Materials

The following supporting information can be downloaded at https://www.mdpi.com/article/10.3390/jcm15207911/s1: Supplementary File S1: Study Protocol for “Simulation-Based Training Tools for Cleft Lip Repair: A Systematic Review”; Supplementary File S2: PRISMA 2020 and PRISMA 2020 for Abstracts Checklists; Supplementary File S3: PICO Framework; Supplementary File S4: Complete Search Strategies; Supplementary File S5: Publication-Level Data Extraction and Full-Text Eligibility Decisions; Supplementary File S6: Publication-Level Messick Validity Evidence and McGaghie Educational Effectiveness Ratings and Rationale; Supplementary File S7: Mixed Methods Appraisal Tool (MMAT) Assessment. Reports assessed in full text but excluded from the review are cited here [57,58,59,60,61,62,63,64,65,66,67,68,69,70]. Detailed reasons for exclusion are provided in Supplementary File S5.

## References

[1] Salari N., Darvishi N., Heydari M., Bokaee S., Darvishi F., Mohammadi M. (2022). Global prevalence of cleft palate, cleft lip and cleft palate and lip: A comprehensive systematic review and meta-analysis. J. Stomatol. Oral Maxillofac. Surg..

[2] Kamiloglu B., Radwan M.T. (2026). Genetic and Molecular Basis of Cleft Lip and Palate: A Comprehensive Review. Diagnostics.

[3] Guarino M. (2025). The developmental bases of cleft lip and cleft palate: Cellular and molecular mechanisms. Anat. Cell Biol..

[4] Shkoukani M.A., Chen M., Vong A. (2013). Cleft Lip—A Comprehensive Review. Front. Pediatr..

[5] Yuzuriha S., Mulliken J.B. (2008). Minor-Form, Microform, and Mini-Microform Cleft Lip: Anatomical Features, Operative Techniques, and Revisions. Plast. Reconstr. Surg..

[6] Jula Cazacu C., Jula C.R., Grammatikis E., Șapte E. (2026). Quality of life in patients with craniofacial anomalies: Personal experience and review of literature. Med. Pharm. Rep..

[7] Lethaus B., Grau E., Kloss-Brandstätter A., Brauer L., Zimmerer R., Bartella A.K., Hahnel S., Sander A.K. (2021). Clinical Follow-Up in Orofacial Clefts—Why Multidisciplinary Care Is the Key. J. Clin. Med..

[8] Anastassov G.E., Joos U. (2001). Comprehensive management of cleft lip and palate deformities. J. Oral Maxillofac. Surg..

[9] Oh T.S., Kim Y.C. (2023). A comprehensive review of surgical techniques in unilateral cleft lip repair. Arch. Craniofacial Surg..

[10] Sayadi L.R., Hamdan U.S., Zhangli Q., Vyas R.M. (2022). Harnessing the Power of Artificial Intelligence to Teach Cleft Lip Surgery. Plast. Reconstr. Surg.—Glob. Open.

[11] Tan S.P.K., Ganske I.M., Mulliken J.B. (2020). Nasolabial Revisions in Unilateral Incomplete Cleft Lip: One Surgeon’s 28-Year Experience. Plast. Reconstr. Surg..

[12] Lastoria D.A.A., Rehman S., Ahmed F., Jasionowska S., Salibi A., Cavale N., Dasgupta P., Aydin A. (2025). A Systematic Review of Simulation-Based Training Tools in Plastic Surgery. J. Surg. Educ..

[13] Ahmed N., Devitt K.S., Keshet I., Spicer J., Imrie K., Feldman L., Cools-Lartigue J., Kayssi A., Lipsman N., Elmi M. (2014). A Systematic Review of the Effects of Resident Duty Hour Restrictions in Surgery: Impact on Resident Wellness, Training, and Patient Outcomes. Ann. Surg..

[14] Braza M.E., Adams N.S., Ford R.D. (2020). Perceptions of Preparedness in Plastic Surgery Residency Training. Plast. Reconstr. Surg.—Glob. Open.

[15] Podolsky D.J., Wong Riff K.W., Drake J.M., Forrest C.R., Fisher D.M. (2018). A High Fidelity Cleft Lip Simulator. Plast. Reconstr. Surg.—Glob. Open.

[16] Badash I., Burtt K., Solorzano C.A., Carey J.N. (2016). Innovations in surgery simulation: A review of past, current and future techniques. Ann. Transl. Med..

[17] Khoo R., Ryan E., Vijayasekaran V., Gillet D.A. (2023). Cleft lip repair simulation: An Australian trainee experience. Australas. J. Plast. Surg..

[18] Meara J.G., Leather A.J.M., Hagander L., Alkire B.C., Alonso N., Ameh E.A., Bickler S.W., Conteh L., Dare A.J., Davies J. (2015). Global Surgery 2030: Evidence and solutions for achieving health, welfare, and economic development. Lancet.

[19] Lutz R., Kesting M.R., Weber M., Olmos M., Tasyürek D., Möst T., Bürstner J., Schulz K.L. (2023). An ex vivo model for education and training of bilateral cleft lip surgery. BMC Med. Educ..

[20] Reighard C.L., Green K., Rooney D.M., Zopf D.A. (2019). Development of a Novel, Low-Cost, High-fidelity Cleft Lip Repair Surgical Simulator Using Computer-Aided Design and 3-Dimensional Printing. JAMA Facial Plast. Surg..

[21] Rogers-Vizena C.R., Saldanha F.Y.L., Hosmer A.L., Weinstock P.H. (2018). A New Paradigm in Cleft Lip Procedural Excellence: Creation and Preliminary Digital Validation of a Lifelike Simulator. Plast. Reconstr. Surg..

[22] Martin J.A., Regehr G., Reznick R., Macrae H., Murnaghan J., Hutchison C., Brown M. (1997). Objective structured assessment of technical skill (OSATS) for surgical residents: Objective Structured Assessment of Technical Skill. Br. J. Surg..

[23] Rogers-Vizena C.R., Yao C.A., Sideridis G.D., Minahan L.B., Saldanha F.Y.L.M., Livingston K.A.M., Weinstock P.H. (2022). Cleft Lip Repair Competence Can Be Evaluated with High-fidelity Simulation. Plast. Reconstr. Surg.—Glob. Open.

[24] Chang B., Powell A., Ellsperman S., Wehrmann D., Landry A., Jabbour N., Goudy S., Zopf D. (2020). Multicenter Advanced Pediatric Otolaryngology Fellowship Prep Surgical Simulation Course with 3D Printed High-Fidelity Models. Otolaryngol. Neck Surg..

[25] Dawe S.R., Pena G.N., Windsor J.A., Broeders J.A.J.L., Cregan P.C., Hewett P.J., Maddern G.J. (2014). Systematic review of skills transfer after surgical simulation-based training. Br. J. Surg..

[26] Kantar R.S., Alfonso A.R., Ramly E.P., Diaz-Siso J.R., Breugem C.C., Flores R.L. (2019). Simulation in Cleft Surgery. Plast. Reconstr. Surg.—Glob. Open.

[27] Raveendran M. (2020). Simulation-Based Training Models for Cleft Palate Repair: A Systematic Review. Cleft Palate Craniofacial J..

[28] Page M.J., McKenzie J.E., Bossuyt P.M., Boutron I., Hoffmann T.C., Mulrow C.D., Shamseer L., Tetzlaff J.M., Akl E.A., Brennan S.E. (2021). The PRISMA 2020 statement: An updated guideline for reporting systematic reviews. BMJ.

[29] Ouzzani M., Hammady H., Fedorowicz Z., Elmagarmid A. (2016). Rayyan—A web and mobile app for systematic reviews. Syst. Rev..

[30] Belur J., Tompson L., Thornton A., Simon M. (2021). Interrater Reliability in Systematic Review Methodology: Exploring Variation in Coder Decision-Making. Sociol. Methods Res..

[31] Cook D.A., Zendejas B., Hamstra S.J., Hatala R., Brydges R. (2014). What counts as validity evidence? Examples and prevalence in a systematic review of simulation-based assessment. Adv. Health Sci. Educ..

[32] Beckman T.J., Cook D.A., Mandrekar J.N. (2005). What is the validity evidence for assessments of clinical teaching?. J. Gen. Intern. Med..

[33] McGaghie W.C., Draycott T.J., Dunn W.F., Lopez C.M., Stefanidis D. (2011). Evaluating the Impact of Simulation on Translational Patient Outcomes. Simul. Healthc. J. Soc. Simul. Healthc..

[34] Hong Q.N., Fàbregues S., Bartlett G., Boardman F., Cargo M., Dagenais P., Gagnon M.-P., Griffiths F., Nicolau B., O’cAthain A. (2018). The Mixed Methods Appraisal Tool (MMAT) version 2018 for information professionals and researchers. Educ. Inf..

[35] Rogers-Vizena C.R., Saldanha F.Y., Sideridis G.D., Allan C.K., Livingston K.A., Nussbaum L., Weinstock P.H. (2023). High-Fidelity Cleft Simulation Maintains Improvements in Performance and Confidence: A Prospective Study. J. Surg. Educ..

[36] Kantar R.S., Gonchar M., Maliha S.G., Ramly E.P., Alfonso A.R., Cohen O., Eisemann B.S., Diaz-Siso J.R., Saadeh P.B., Flores R.L. (2019). Three-Dimensional Affordable Stone Models for Cleft Lip Markings: A Prospective Study of Learner Satisfaction. Ann. Plast. Surg..

[37] Kantar R.S., Alfonso A.R., Ramly E.P., Cohen O., Rifkin W.J., Maliha S.G., Diaz-Siso J.R., Eisemann B.S., Saadeh P.B., Flores R.L. (2020). Knowledge and Skills Acquisition by Plastic Surgery Residents through Digital Simulation Training: A Prospective, Randomized, Blinded Trial. Plast. Reconstr. Surg..

[38] Lutz R., Schulz K.L., Weber M., Olmos M., Möst T., Bürstner J., Kesting M.R. (2023). An ex vivo model for education and training of unilateral cleft lip surgery. BMC Med. Educ..

[39] Zaga-Galante J., Tse R., Hopper R.A., Arnold A., Fisher D.M., Wong-Riff K.W., Podolsky D.J. (2025). Bilateral Cleft lip Simulation. Cleft Palate Craniofacial J..

[40] Vogl C., Schulz K.L., Olmos M., Weber M., Möst T., Kesting M.R., Lutz R. (2025). Unilateral and bilateral ex vivo cleft lip models: Universal tools for surgical training at different levels of experience. Head Face Med..

[41] Wang X.L., Xie Y., Yue Y.X., Yang X.W., Li J. (2025). A novel paradigm in cleft lip education: Integration of 3D-printed simulator and problem-based learning. BMC Med. Educ..

[42] Riedle H., Burkhardt A.E., Seitz V., Pachaly B., Reid R.R., Lee J.C., Franke J.E. (2019). Design and fabrication of a generic 3D-printed silicone unilateral cleft lip and palate model. J. Plast. Reconstr. Aesthet. Surg..

[43] Kantar R.S., Plana N.M., Cutting C.B., Diaz-Siso J.R., Flores R.L. (2018). Internet-Based Digital Simulation for Cleft Surgery Education: A 5-Year Assessment of Demographics, Usage, and Global Effect. J. Surg. Educ..

[44] Saldanha F.Y.L., Loan G.J., Calabrese C.E., Sideridis G.D., Weinstock P.H., Rogers-Vizena C.R. (2021). Incorporating Cleft Lip Simulation Into a “Bootcamp-Style” Curriculum. Ann. Plast. Surg..

[45] Lobos C.T., Benitez B.K., Lill Y., Kiser L.E., Tache A., Fernandez-Pose M., Gonzalez A.C., Nalabothu P., Sharma N., Thieringer F.M. (2024). Cleft lip and palate surgery simulator: Open source simulation model. Heliyon.

[46] Kaimi P., Fisher D.M., Yasabala B., Wong Riff K.W., Podolsky D.J. (2025). Technical skills assessment during simulated cleft lip repair. J. Plast. Reconstr. Aesthet. Surg..

[47] Chen Y., Li M., Wu Y., Wang L., Cui Q. (2024). Design and fabrication of silicone cleft lip simulation model for personalized surgical training. J. Plast. Reconstr. Aesthet. Surg..

[48] Volk A.S., Eisemann B.S., Dibbs R.P., Perdanasari A.T., Braun T.L., Marsack K.P. (2020). The Utility of an Open-Access Surgical Simulator to Enhance Surgeon Training. J. Craniofacial Surg..

[49] Michaels R.E., Zugris N.V., Cin M.D., Monovoukas D.A., Koka K.K., Smith C., Bohm L., Kim J., Green G., Roby B. (2022). A national pediatric otolaryngology fellowship virtual dissection course using 3D printed simulators. Int. J. Pediatr. Otorhinolaryngol..

[50] Stanek K., Silver M., Edman A., Nussbaum L., Weinstock P.H., Rogers-Vizena C.R. (2025). Gauging the Optimal Simulator Complexity for Learning Cleft Lip Repair: A Cost-Benefit Analysis. Plast. Reconstr. Surg.—Glob. Open.

[51] Plana N.M., Rifkin W.J., Kantar R.S., David J.A., Maliha S.G., Farber S.J., Staffenberg D.A., Grayson B.H., Diaz-Siso J.R., Flores R.L. (2019). A Prospective, Randomized, Blinded Trial Comparing Digital Simulation to Textbook for Cleft Surgery Education. Plast. Reconstr. Surg..

[52] Kotsis S.V., Chung K.C. (2013). Application of the “See One, Do One, Teach One” Concept in Surgical Training. Plast. Reconstr. Surg..

[53] Aydin A., Raison N., Khan M.S., Dasgupta P., Ahmed K. (2016). Simulation-based training and assessment in urological surgery. Nat. Rev. Urol..

[54] Minha S., Shefet D., Sagi D., Berkenstadt H., Ziv A. (2016). “See One, Sim One, Do One”—A National Pre-Internship Boot-Camp to Ensure a Safer “Student to Doctor” Transition. PLoS ONE.

[55] D’aBbondanza J.A., Shih J.G., Knox A.D.C., Zhygan N., Brown M.H., Fish J.S., Courtemanche D.J. (2024). Resident Exposure and Involvement in Core Procedural Competencies within Pediatric Plastic Surgery. Plast. Surg..

[56] Foppiani J., Stanek K., Alvarez A.H., Weidman A., Valentine L., Oh I.J., Albakri K., Choudry U., Rogers-Vizena C.R., Lin S.J. (2024). Merits of simulation-based education: A systematic review and meta-analysis. J. Plast. Reconstr. Aesthet. Surg..

[57] Borg T.-M., Solomon S., Alfarrouh R., Barazi W., Abu Sittah G., Sommerlad B., Ghanem A. (2024). Simulation Training Approach for Cleft Lip and Palate Repair in Low-Income Countries. Cleft Palate Craniofacial J..

[58] Ueda K., Kino H., Katayama M., Hirota Y. (2020). Simulation Surgery Using 3D 3-layer Models for Congenital Anomaly. Plast. Reconstr. Surg.—Glob. Open.

[59] Rogers-Vizena C.R., Sideridis G.D., Patel K.G., Garland C.B., Mount D.L., Yao C.A. (2020). A Competency Assessment Tool for Unilateral Cleft Lip Repair. Plast. Reconstr. Surg.—Glob. Open.

[60] Dalgalı P., Topsakal K.G., Eser Mısır S., Samur Ergüven S., Duran G.S., Görgülü S. (2024). Evaluating the Impact of Different Education Methods on Cleft Lip and Palate Anatomy Training. Cleft Palate Craniofacial J..

[61] AlAli A.B., Griffin M.F., Calonge W.M., Butler P.E. (2018). Evaluating the Use of Cleft Lip and Palate 3D-Printed Models as a Teaching Aid. J. Surg. Educ..

[62] Taylor J., Abramowicz S., Goudy S., Michaels R., Zopf D., Ruiz R., Tiwana P. A Primer Course for Pediatric Cleft and Craniomaxillofacial Surgery Fellows. Proceedings of the ACPA 80th Annual Meeting Program Guide.

[63] Kalmar C., Plana N., Carlson A., Zapatero Z., Kosyk M., Taylor J., Swanson J. (2021). Interactive Touchscreen Training Simulator for Surgical Incision Markings for Cleft Lip Repair. Cleft Palate Craniofacial J..

[64] Cutting C., Sifakis E., Wang Q., Tao Y., Flores R. (2021). A computer-based simulator for the study of unilateral cleft lip repair. Cleft Palate Craniofacial J..

[65] Rogers-Vizena C.R., Yao C.A., Sideridis G.D., Minahan L., Saldanha F.Y.L., Livingston K.A., Weinstock P.H. (2021). Evaluating competence with cleft lip repair using high fidelity simulation. Cleft Palate Craniofacial J..

[66] Kantar R.S., Gonchar M., Maliha S.G., Ramly E.P., Alfonso A.R., Eisemann B.S., Shetye P.R., Grayson B.H., Saadeh P.B., Flores R.L. (2019). Learner Satisfaction with Three-Dimensional Affordable Stone Models for Cleft Lip Markings: Results from a Prospective Study. Cleft Palate Craniofacial J..

[67] Diaz-Siso J.R., Kantar R., Rifkin W., Plana N., David J., Maliha S., Flores R. (2018). A prospective, randomized, blinded trial in cleft surgery training: Textbook vs. digital simulation. Cleft Palate Craniofacial J..

[68] Podolsky D.J., Riff K.W.W., Drake J.M., Forrest C.R., Fisher D.M. (2017). Development and Evaluation of a Novel High-Fidelity Cleft Lip Simulator. Cleft Palate Craniofacial J..

[69] Stanek K., Yao C.A., Livingston K.A., Weinstock P.H., Rogers-Vizena C.R. (2024). Teaching Unilateral Cleft lip Repair: Lessons from Simulation-Based Mining of Trainee Strengths and Performance Gaps. Cleft Palate Craniofacial J..

[70] Vyas R.M., Sayadi L.R., Bendit D., Hamdan U.S. (2020). Using Virtual Augmented Reality to Remotely Proctor Overseas Surgical Outreach: Building Long-Term International Capacity and Sustainability. Plast. Reconstr. Surg..

