# Supplementary material for: Simulation-Based Training Tools for Cleft Lip Repair: A Systematic Review
Source: J Clin Med. 2026 Oct 13;15(20):7911. doi: 10.3390/jcm15207911 (PMC13706072; doi:10.3390/jcm15207911)
Supplement: Supplementary file 1 [file jcm-15-07911-s001.zip › Supplementary File S1_ Study protocol Simulation-Based Training Tools for Cleft Lip Repair_ A Systematic Review_.pdf]

Study protocol

Article title: *Simulation-Based Training Tools for Cleft Lip Repair: A Systematic Review*

Objective:
The aim of this systematic review is to identify and characterize simulators, task trainers, and training models used for surgical education and training in cleft lip repair. A secondary aim is to synthesize the available evidence on their design and construction, realism, validity, usability, and educational effectiveness.

Methods:

1. **Search Strategy and Selection Criteria**

A comprehensive literature search was conducted in the following databases: MEDLINE via PubMed, Scopus, Web of Science Core Collection, and Embase.
The search strategy was developed according to the PICO framework and included two main concepts:
surgical treatment of cleft lip;
simulators, task trainers, and training models used in surgical education and training.
Terms within each concept were combined using the Boolean operator OR, while the two main concepts were combined using AND. Appropriate controlled vocabulary terms, including MeSH terms in PubMed and Emtree terms in Embase, were used where applicable. The search strategy was adapted to the syntax and indexing system of each database.

The search was limited to publications published between 2016 and 2026. No restrictions regarding the type of simulation model or the level of experience of its users were applied at the search stage.

2. **Inclusion and exclusion criteria**

Inclusion Criteria

Studies were eligible for inclusion if they:

- were original research articles;
- described simulators, task trainers, training models, or other tools intended for education or surgical training in cleft lip repair;
- concerned unilateral or bilateral cleft lip, primary cleft lip repair, cheiloplasty, or a clearly defined stage of the procedure;
- described the development, construction, evaluation, validation, usability, or educational effectiveness of a training tool;
- involved physical, synthetic, silicone, three-dimensionally printed, biological, cadaveric, animal-based, virtual, haptic, or hybrid models;
- provided sufficient information regarding the construction, evaluation, validation, usability, or educational effectiveness of the training tool;
- were available as full-text publications in English.

Studies addressing both cleft lip and cleft palate were eligible only when the component related to cleft lip repair training was clearly described and the relevant data could be extracted separately.

Exclusion Criteria

Studies were excluded if they:

- concerned cleft palate, alveolar clefts, alveolar bone grafting, or other craniofacial clefts without a clearly identifiable component related to cleft lip repair;
- described general suturing, microsurgical, plastic surgery, or reconstructive surgery models without a specific application to cleft lip repair;

- focused exclusively on preoperative planning, anatomical visualization, simulation of treatment outcomes, intraoperative navigation, or preparation of a patient-specific procedure without an educational or training component;
- described models intended exclusively for anatomical demonstration, diagnosis, patient counselling, or clinical treatment;
- presented only a theoretical concept or proposed design of a training tool without its construction, implementation, practical use, or evaluation;
- used a cleft lip simulator or training model solely as a platform for investigating another research question, without evaluating the training tool itself or its educational effectiveness;
- were case reports, systematic reviews, scoping reviews, meta-analyses, narrative reviews, protocols, guidelines, commentaries, letters to the editor, or expert opinions;
- were conference abstracts without an available full-text publication;
- were published before 2016 or in a language other than English.

3. **Study selection**

The study selection process was conducted by two independent reviewers. After duplicate records had been removed, both reviewers independently screened the titles and abstracts in accordance with the predefined eligibility criteria. Full texts of potentially relevant publications were then retrieved and independently assessed for eligibility.

Conflicting decisions at either stage of the selection process were adjudicated by a third reviewer. Reasons for exclusion at the full-text stage were recorded. The study selection process was presented using the PRISMA 2020 flow diagram.

4. **Data Extraction**

Data from studies included in the review will be extracted using a standardized and pilot-tested data extraction form. Data from each publication will be independently extracted by two reviewers. Any discrepancies will be resolved through discussion, and if consensus cannot be reached, a third reviewer will make the final decision.

The following information will be extracted from the included studies:

- authors, year of publication, country, journal, and study objective;
- study type and design;
- number and characteristics of participants, including their level of surgical experience;
- type of cleft lip and the surgical technique or stage of the procedure addressed by the training;
- type of simulator, task trainer, or training model;
- materials and technologies used to construct the model;
- training format, number of sessions, and duration of training;
- methods used to assess and validate the training tool;
- anatomical and procedural realism, usability, and user satisfaction;
- educational outcomes, including changes in knowledge, confidence, and technical skills;
- procedural accuracy, task completion time, and number of errors, when assessed;
- cost, durability, availability, and reusability of the model;
- study limitations, sources of funding, and declared conflicts of interest.

5. **Checklists**
   a. PRISMA 2020 checklist
   b. PRISMA 2020 abstract checklist
   c. PICO

6. **Methodological quality and risk of bias assessment**

The methodological quality and risk of bias of the included studies will be assessed independently by two reviewers. Appropriate critical appraisal tools will be selected according to the design of each included study. Where applicable, JBI critical appraisal checklists will be used for randomized, quasi-experimental, observational, and cross-sectional studies. Any disagreements will be resolved through discussion, and if consensus cannot be reached, a third reviewer will make the final decision.

7. Funding

This systematic review received no external funding.

8. Protocol Amendments

The protocol was first submitted to PROSPERO on 5 August 2026 and registered on 11 August 2026, after study screening and during data extraction. Following registration, the methodological quality appraisal approach was amended. The original protocol specified the use of design-specific critical appraisal tools, including JBI checklists where applicable. The final review used the Mixed Methods Appraisal Tool (MMAT), version 2018, to provide a consistent methodological quality assessment framework across the heterogeneous study designs included in the review. This amendment was implemented before the formal methodological quality assessment of the included studies and was reflected in PROSPERO version 1.1, published on 12 September 2026. No included study had been formally appraised using the originally planned design-specific tools before the amendment. This methodological change did not affect the search strategy, eligibility criteria, study selection process, or data extraction procedures.

The Messick validity framework and the adapted McGaghie educational effectiveness framework were subsequently introduced to structure the evidence synthesis and were selected before the original independent study-level ratings were assigned. During major revision, their operational criteria were clarified and the final judgments were re-examined. During minor revision, both reviewers independently reassessed all included publications using the final operational criteria. Percentage agreement and unweighted Cohen's κ were recalculated from these updated independent ratings before consensus adjudication. The final consensus classifications remained unchanged. These refinements did not change the set of included publications.
