# Supplementary material for: Simulation-Based Training Tools for Cleft Lip Repair: A Systematic Review
Source: J Clin Med. 2026 Oct 13;15(20):7911. doi: 10.3390/jcm15207911 (PMC13706072; doi:10.3390/jcm15207911)
Supplement: Supplementary file 1 [file jcm-15-07911-s001.zip › Supplementary File S3_ PICO Framework.pdf]

PICO scheme of searching

**PICO elements**	**Keywords**	**Search terms**	**Search strategy**
**P** (**P**atient or/and **P**opulation)	Surgical education and training in cleft lip repair	Cleft Lip, Cleft Lip Repair, Cheiloplasty, Primary Lip Repair, Unilateral Cleft Lip, Bilateral Cleft Lip	Cleft Lip OR Cleft Lip Repair OR Cheiloplast* OR Primary Lip Repair OR Unilateral Cleft Lip OR Bilateral Cleft Lip
**I** (**I**ntervention)	Simulation-based training tools and surgical models	Simulation Training, Models, Anatomic, Printing, Three-Dimensional, Virtual Reality, Augmented Reality, Haptic Technology, Surgical Simulator, Training Model, Task Trainer	Simulation Training OR Models, Anatomic OR Printing, Three-Dimensional OR Virtual Reality OR Augmented Reality OR Haptic Technology OR Simulat* OR Simulator* OR Surgical Simulator OR Training Model OR Surgical Model OR Task Trainer OR Physical Model OR Synthetic Model OR 3D Print*
**C** (**C**omparison)	Not applicable	Not applicable	Not applicable
**O** (**O**utcome)	Characteristics, validity, usability and educational effectiveness of training tools	Clinical Competence, Educational Measurement, Technical Skills, Performance, Accuracy, Realism, Fidelity, Validity, Usability	Not applicable
