# Supplementary material for: Simulation-Based Training Tools for Cleft Lip Repair: A Systematic Review
Source: J Clin Med. 2026 Oct 13;15(20):7911. doi: 10.3390/jcm15207911 (PMC13706072; doi:10.3390/jcm15207911)
Supplement: Supplementary file 1 [file jcm-15-07911-s001.zip › Supplementary File S4_ Complete Search Strategies.pdf]

Search strategy (01 August 2026)

Conceptual search framework
(Cleft Lip OR Cleft Lip Repair OR Cleft Lip Surgery OR Cheiloplast* OR Labioplast* OR Primary Lip Repair OR Unilateral Cleft Lip OR Bilateral Cleft Lip) AND (Simulation Training OR Simulation-Based Training OR Simulat* OR Simulator* OR Surgical Simulator* OR Surgical Trainer* OR Task Trainer* OR Training Model* OR Surgical Model* OR Physical Model* OR Synthetic Model* OR Anatomic Model* OR Anatomical Model* OR 3D Print* OR Three-Dimensional Print* OR Virtual Reality OR Augmented Reality OR Mixed Reality OR Haptic*)

Date restrictions were applied at the database-search stage. No language restriction was applied during database searching; the English-language restriction was applied during study screening according to the predefined eligibility criteria.

PubMed

Original search date: 1 August 2026
Original date filter: Last 10 years
Date-range verification/re-run: 23 September 2026
Standardized date limits: 1 January 2016 to 1 August 2026
Search fields: MeSH terms and Title/Abstract ([tiab])
Controlled vocabulary: MeSH
Language restriction: None applied at the database-search stage
Other filters: None
("Cleft Lip"[Mesh] OR "cleft lip"[tiab] OR "cleft lip repair"[tiab] OR "cleft lip surgery"[tiab] OR cheiloplast*[tiab] OR labioplast*[tiab] OR "primary lip repair"[tiab] OR "unilateral cleft lip"[tiab] OR "bilateral cleft lip"[tiab]) AND ("Simulation Training"[Mesh] OR simulat*[tiab] OR "surgical simulator*"[tiab] OR "surgical trainer*"[tiab] OR "task trainer*"[tiab] OR "training model*"[tiab] OR (("Models, Anatomic"[Mesh] OR "Printing, Three-Dimensional"[Mesh] OR "Virtual Reality"[Mesh] OR "Augmented Reality"[Mesh] OR "Haptic Technology"[Mesh] OR "Haptic Interfaces"[Mesh] OR "surgical model*"[tiab] OR "physical model*"[tiab] OR "synthetic model*"[tiab] OR "3D print*"[tiab] OR "three-dimensional print*"[tiab] OR "virtual reality"[tiab] OR "augmented reality"[tiab] OR "mixed reality"[tiab] OR haptic*[tiab]) AND (educat*[tiab] OR train*[tiab] OR teach*[tiab] OR learn*[tiab] OR skill*[tiab])))
Results before date filtering: 289
Results after filtering: 205

Original results after filtering: 200
Results after standardized date filtering: 205
The PubMed search was re-run during revision using the standardized date range. Five additional records were retrieved; all were duplicates of records already identified through the other database searches.

Scopus:

Platform: Scopus (Elsevier)
Search date: 1 August 2026
Search fields: Title, Abstract, Keywords (`TITLE-ABS-KEY`)
Controlled vocabulary: Not applicable
Date limits: Publication years 2016–2026, inclusive
Language restriction: None applied at the database-search stage
Other filters: None

TITLE-ABS-KEY(
("cleft lip" OR "cleft lip repair" OR "cleft lip surgery" OR cheiloplast* OR labioplast* OR "primary lip repair" OR "unilateral cleft lip" OR "bilateral cleft lip")
AND

("simulation training" OR "simulation-based training" OR simulat* OR simulator* OR "surgical simulator" OR "surgical trainer" OR "task trainer" OR "training model" OR "surgical model" OR "physical model" OR "synthetic model" OR "3D print" OR "three-dimensional print" OR "virtual reality" OR "augmented reality" OR "mixed reality" OR haptic*
OR
(("anatomic model" OR "anatomical model" OR "3D printing" OR "three-dimensional printing")
AND
(educat* OR train* OR teach* OR learn* OR skill*)))
)

Results before filtering: 413
Results after filtering: 284

Web of Science:

Platform: Web of Science Core Collection (Clarivate)
Search date: 1 August 2026
Search fields: Topic (`TS`)
Controlled vocabulary: Not applicable
Date limits: Publication years 2016–2026, inclusive
Language restriction: None applied at the database-search stage
Other filters: None

TS=((“cleft lip” OR “cleft lip repair” OR “cleft lip surgery” OR cheiloplast* OR labioplast* OR “primary lip repair” OR “unilateral cleft lip” OR “bilateral cleft lip”) AND (“simulation training” OR “simulation-based training” OR simulat* OR simulator* OR “surgical simulator*” OR “surgical trainer*” OR “task trainer*” OR “training model*” OR ((“surgical model*” OR “physical model*” OR “synthetic model*” OR “anatomic model*” OR “anatomical model*” OR “3D print*” OR “three-dimensional print*” OR “virtual reality” OR “augmented reality” OR “mixed reality” OR haptic*) AND (educat* OR train* OR teach* OR learn* OR skill*))))

Results before filtering: 286
Results after filtering: 221

Embase:

Platform: Embase.com
Search date: 1 August 2026
Search fields: Title, Abstract, Keywords (`:ti,ab,kw`)
Controlled vocabulary: Emtree, exploded terms (`/exp`)
Date limits: Publication years 2016–2026, inclusive
Language restriction: None applied at the database-search stage
Other filters: Document type: Article, Review

('cleft lip'/exp OR 'cleft lip':ti,ab,kw OR 'cleft lip repair':ti,ab,kw OR 'cleft lip surgery':ti,ab,kw OR cheiloplast*:ti,ab,kw OR labioplast*:ti,ab,kw OR 'primary lip repair':ti,ab,kw OR 'unilateral cleft lip':ti,ab,kw OR 'bilateral cleft lip':ti,ab,kw) AND ('simulation training'/exp OR 'simulation training':ti,ab,kw OR 'simulation-based training':ti,ab,kw OR simulat*:ti,ab,kw OR simulator*:ti,ab,kw OR 'surgical simulator*':ti,ab,kw OR 'surgical trainer*':ti,ab,kw OR 'task trainer*':ti,ab,kw OR 'training model*':ti,ab,kw OR (('anatomical model'/exp OR 'three dimensional printing'/exp OR 'virtual reality'/exp OR 'augmented reality'/exp OR 'surgical model*':ti,ab,kw OR 'physical model*':ti,ab,kw OR 'synthetic model*':ti,ab,kw OR 'anatomic model*':ti,ab,kw OR 'anatomical model*':ti,ab,kw OR '3d print*':ti,ab,kw OR 'three-dimensional print*':ti,ab,kw OR 'virtual reality':ti,ab,kw OR 'augmented reality':ti,ab,kw OR 'mixed reality':ti,ab,kw OR haptic*:ti,ab,kw) AND (educat*:ti,ab,kw OR train*:ti,ab,kw OR teach*:ti,ab,kw OR learn*:ti,ab,kw OR skill*:ti,ab,kw)))

Results before filtering: 335

Results after filtering: 254

Results of search summary:

Records identified after database-specific filters: 964
Duplicate records removed: 575
First scanning: 389
The conflicts between researchers: 32
Researcher 1: excluded 357, included 32
Researcher 2: excluded 333, included 56
Records excluded after title/abstract screening: 351
Articles accepted for retrieval: 38
Reports excluded after full-text assessment: 14
Conference abstract/meeting supplement (n = 7)
No eligible simulation-based cleft lip surgical training intervention (n = 4)
Simulator used solely as a research/assessment platform (n = 2)
Cleft lip-specific outcomes not separately extractable (n = 1)
Articles included in study: 24

Results:
Cohen's k: 0.594
