# Supplementary material for: Simulation-Based Training Tools for Cleft Lip Repair: A Systematic Review
Source: J Clin Med. 2026 Oct 13;15(20):7911. doi: 10.3390/jcm15207911 (PMC13706072; doi:10.3390/jcm15207911)
Supplement: Supplementary file 1 [file jcm-15-07911-s001.zip › Supplementary File S6_ Publication-Level Messick Validity Evidence and McGaghie Educational Effectiveness Ratings and Rationale.pdf]

Table S1. Operational criteria used in this review to apply the N/0/1/2 validity-evidence rating structure adapted from Beckman et al.

Messick domain	N - Not assessable	0 - Not supportive	1 - Limited evidence	2 - Strong evidence
Content	No relevant evidence addressing whether the content of a defined assessment score, instrument, or observation adequately represented the intended construct or procedure.	The content of a defined assessment score, instrument, or observation was evaluated, but the findings did not support adequate representation of the intended construct or procedure.	Limited, indirect, or partial evidence that the items, scoring criteria, or observed performance components of a defined assessment represented the intended construct or procedure, such as limited expert review or incomplete content mapping.	Strong, direct, and methodologically robust evidence that the content of a defined assessment instrument, score, or observation represented the intended construct or procedure, such as structured expert consensus, cognitive task analysis, systematic content mapping, or independent expert review specifically addressing the assessment instrument or scoring criteria.
Response Process	No relevant evidence on standardization of administration, scoring, or rater procedures.	Response processes were evaluated, but procedures did not support the intended interpretation.	Partial standardization of administration or scoring.	Multiple or strong safeguards against response or scoring error, such as standardized administration, blinded or independent scoring, formal rater calibration, analysis of rater effects, or other procedures demonstrating consistent scoring.
Internal Structure	No reliability or internal-consistency analysis was reported.	Reliability/internal structure was assessed but findings did not support adequate consistency.	Limited, incomplete, or mixed reliability evidence.	Strong structural or reliability evidence, including multiple complementary reliability analyses, confirmed internal structure, or consistently strong reliability across raters, occasions, or complementary measures.
Relations to Other Variables	No relevant relationship with an external variable was examined.	An expected relationship was examined but not demonstrated.	Limited or partial association with a relevant external variable.	Strong and direct relationship with a relevant external variable, such as clear discrimination by expertise level or association with objective performance

				measures.
Consequences	No evidence regarding intended or unintended consequences of using assessment results, including proficiency thresholds, pass/fail decisions, progression criteria, or other downstream consequences of score interpretation.	Consequences were evaluated but did not support the intended use.	Limited or preliminary evidence regarding the consequences of assessment use, such as a proposed proficiency threshold, pass/fail criterion, or progression rule without formal validation or without sufficient evidence supporting its intended application.	Strong and direct empirical evidence that the use of assessment results produced appropriate intended consequences and/or systematically evaluated relevant unintended consequences.

Note. The N/0/1/2 rating structure was adapted from Beckman et al., with domain-specific operational criteria refined for the simulation-based context of the present review. Ratings were assigned independently for each domain and were not summed into an overall validity score. Ratings reflected evidence supporting the intended interpretation of simulator- or assessment-derived observations or scores rather than overall study quality. Ratings were based exclusively on evidence explicitly reported in the included studies. Anatomical realism, procedural fidelity, perceived usefulness, satisfaction, expert teaching, and the organization of training were not considered evidence of assessment validity by themselves unless they directly supported the intended interpretation of a defined simulator- or assessment-derived score or observation.

Table S2. Independent reviewer ratings and final consensus for Messick validity evidence

ID	Publication	Score/observation evaluated	Intended interpretation	Content	Response Process	Internal Structure	Relations to Other Variables	Consequences	Domain-specific supporting evidence and rationale
1	Zaga-Galante et al.	Expert questionnaire ratings of simulator characteristics and representation of relevant anatomical/procedural features.	More favorable expert ratings were intended to indicate better representation of relevant cleft anatomy and procedural content by the simulator, rather than trainee competence.	1 / 1 / 1	1 / 1 / 1	N / N / N	N / N / N	N / N / N	An expert-developed questionnaire completed by experienced cleft surgeons provided limited evidence that the evaluated simulator characteristics represented the intended procedural content, supporting a Content rating of 1. Structured expert evaluation supported limited Response Process evidence (1). No assessable evidence was reported for Internal Structure, Relations to Other Variables, or Consequences.
2	Lutz et al. - bilateral	Structured participant self-assessment/questionnaire responses concerning bilateral cleft-lip simulation.	Responses were intended to reflect participants' self-perceived confidence, preparedness, or educational experience rather than objectively measured technical competence.	N / 1 / N	1 / 1 / 1	N / N / N	N / N / N	N / N / N	Structured participant assessment supported limited Response Process evidence (1). No assessable evidence was reported for Content, Internal Structure, Relations to Other Variables, or Consequences.
3	Lutz et al. -	Pre/post participant questionnaire or	Changes in responses were intended to reflect	N / N / N	1 / N / 1	N / N / N	N / N / N	N / N / N	Standardized pre/post questionnaire administration supported limited Response

	unilateral	self-assessment responses.	changes in participants' self-perceived confidence/preparedness or educational experience, not objective technical skill.						Process evidence (1). No assessable evidence was reported for Content, Internal Structure, Relations to Other Variables, or Consequences.
4	Vogl et al.	Procedure-related assessment results together with structured expert evaluation of the model.	The procedure-related assessment was intended to reflect performance or understanding of the relevant procedural construct, while expert observations addressed the model's representation of that construct.	0 / 1 / 1	1 / 1 / 1	N / N / N	0 / 0 / 0	N / N / N	Expert evaluation of the model and procedure-related assessment provided limited evidence that the assessed content represented the intended procedural construct, supporting a Content rating of 1. Structured assessment supported limited Response Process evidence (1). Relations to Other Variables was examined through experience-level comparisons, but the expected relationship was not demonstrated, resulting in a rating of 0. No assessable Internal Structure or Consequences evidence was reported.
5	Wang et al.	Theoretical and operative assessment scores based on established examination content with predefined items/scoring standards.	Higher scores were intended to indicate greater theoretical knowledge and/or better operative performance within the simulated educational setting.	1 / 1 / 1	1 / 1 / 1	N / N / N	N / N / N	N / N / N	The operational assessment was based on established examination content with defined items and scoring standards, providing limited Content evidence (1). Standardized scoring procedures provided limited Response Process evidence (1). No assessable evidence was reported for Internal Structure, Relations to Other Variables, or Consequences
6	Riedle et al.	Pre- and post-session questionnaire responses	Responses were intended to reflect participants' perceptions of the simulator and training	N /N / N	1 / 1 / 1	N / N / N	N / N / N	N / N / N	Structured pre- and post-session questionnaire administration supported limited Response Process evidence (1). No assessable evidence was reported for Content, Internal Structure,

		concerning the simulator and training experience, including perceptual characteristics such as realism/usability.	experience before and after the session, not objectively measured surgical competence.						Relations to Other Variables, or Consequences.
7	Kantar et al. - 5-year digital assessment	No defined assessment score used for validity inference. Reported observations primarily concerned platform use/reach and learner perceptions/usefulness.	The reported observations described platform uptake and perceived educational value rather than supporting an inference about a defined knowledge, skill, or performance score.	1 / N / N	1 / N / N	N / N / N	N / N / N	N / N / N	The study primarily evaluated platform use, reach, and learner perceptions rather than evidence supporting the validity of assessment interpretations. Consequently, none of the Messick domains were assessable.
8	Rogers-Vizena et al. - simulator development	Predefined simulator-derived measurements and expert observations, with comparison to patient-derived operative changes.	Simulator-derived observations/measurements were intended to represent clinically relevant anatomical or procedural changes produced by simulated cleft repair and to correspond, at least partially, to patient-derived operative changes.	1 / 1 / 1	1 / 1 / 1	N / N / N	1 / 1 / 1	N / N / N	Expert-informed simulator development and predefined simulator-derived measurements provided limited Content evidence (1). Standardized evaluation supported limited Response Process evidence (1), while comparison with patient-derived operative changes provided limited Relations to Other Variables evidence (1). No assessable Internal Structure or Consequences evidence was reported. .
9	Reighard et al.	Procedure-specific assessment	The instrument was intended to represent and	2 / 2 / 2	1 / 1 / 1	N / N / N	N / N / N	N / N / N	Cognitive task analysis involving cleft surgeons and independent expert review of the assessment

		instrument/observed performance components developed using cognitive task analysis.	assess performance of critical tasks/components of the target cleft procedure.						instrument supported strong Content evidence (2). Structured evaluation supported limited Response Process evidence (1). No assessable evidence was reported for Internal Structure, Relations to Other Variables, or Consequences.
10	Khoo et al.	Standardized pre/post survey responses, principally self-reported confidence.	Higher/post-intervention ratings were intended to reflect greater self-perceived confidence following training rather than objectively demonstrated technical competence.	1 / N / N	1 / N / 1	N / N / N	N / 1 / N	N / N / N	Standardized pre/post surveys supported limited Response Process evidence (1). No assessable evidence was identified for Content, Internal Structure, Relations to Other Variables, or Consequences.
11	Podolsky et al.	Qualitative expert observations by experienced cleft surgeons regarding relevant anatomical and procedural features; no defined numeric assessment score identified in the provided materials.	Expert observations were intended to indicate whether the model represented relevant anatomical and procedural features of the target procedure; they were not interpreted as trainee-performance scores.	1 / 1 / 1	N / N / N	N / N / N	N / N / N	N / N / N	Qualitative evaluation by experienced cleft surgeons provided limited expert evidence regarding the representation of relevant anatomical and procedural features, supporting a Content rating of 1. No assessable evidence was reported for Response Process, Internal Structure, Relations to Other Variables, or Consequences.
12	Saldanha et al.	Self-assessment/confidence responses developed with reference to established training milestones and an	Self-assessment scores were intended to reflect perceived confidence/readiness, while anthropometric observations were intended to reflect	1 / 1 / 1	1 / 1 / 1	N / N / N	1 / 1 / 1	N / N / N	Development of the self-assessment approach with reference to established training milestones and an existing validated confidence instrument provided limited Content evidence (1). Standardized repeated assessment supported limited Response Process evidence (1). Associations between anthropometric outcomes

		existing validated confidence instrument, plus anthropometric outcome measurements.	aspects of the objective procedural result that could be related to training or experience.						and participant training or experience provided limited Relations to Other Variables evidence (1). No assessable Internal Structure or Consequences evidence was reported.
13	Teuber Lobos et al.	Predefined face/content evaluation questionnaire completed by experienced cleft surgeons and trainees.	Questionnaire ratings were intended to characterize how adequately the simulator represented relevant anatomy/procedural characteristics and associated educational features, not to quantify trainee technical competence.	2 / 2 / 2	1 / N / 1	N / N / N	N / N / N	N / N / N	Formal face and content evaluation using a predefined questionnaire completed by experienced cleft surgeons and trainees provided strong Content evidence (2). Use of a standardized questionnaire supported limited Response Process evidence (1). Subjective comparisons across experience levels were insufficient for Relations to Other Variables. No assessable Internal Structure or Consequences evidence was reported.
14	Rogers-Vizena et al. - retention	Procedure-specific technical-performance assessment based on anonymized video recordings, together with objective symmetry-related measurements.	Higher procedure-specific performance scores and favorable objective symmetry measurements were intended to indicate better retained technical performance in simulated cleft repair.	1 / 1 / 1	2 / 2 / 2	N / N / N	1 / 2 / 1	N / N / N	Use of a previously developed procedure-specific assessment supported limited Content evidence (1). Standardized anonymized video assessment with blinded scoring supported strong Response Process evidence (2). Associations of objective performance with training level and symmetry measures provided limited Relations to Other Variables evidence (1). No assessable Internal Structure or Consequences evidence was reported.
15	Rogers-Vizena et al. - competency	Procedure-specific simulated technical-performance score assessed by two	Higher technical-performance scores were intended to indicate greater procedural competence in simulated	1 / 1 / 1	2 / 2 / 2	1 / 1 / 1	2 / 2 / 2	N / N / N	Procedure-specific assessment content supported limited Content evidence (1). Standardized assessment with two blinded independent expert raters supported strong Response Process evidence (2). Reported reliability supported

		blinded independent expert raters, with objective symmetry-related measures.	cleft repair, with objective symmetry measures providing an external indicator of performance quality.						partial Internal Structure evidence (1), while significant associations with training level and objective symmetry measures supported strong Relations to Other Variables evidence (2). No assessable Consequences evidence was reported.
16	Kaimi et al.	Cleft-specific technical assessment scale and global rating scale scores derived from standardized/blinded video ratings of simulated cleft-lip repair.	Higher scores were intended to indicate greater technical proficiency in simulated cleft-lip repair.	2 / 1 / 2	2 / 2 / 2	2 / 2 / 2	1 /2 / 2	N / N / N	Formal expert consensus for scale development supported strong Content evidence (2). Rater calibration and standardized blinded video scoring supported strong Response Process evidence (2). ICC and Cronbach alpha analyses supported strong Internal Structure evidence (2), while discrimination between staff surgeons and trainees supported strong Relations to Other Variables evidence (2). No assessable Consequences evidence was reported.
17	Chen et al.	Objective pre/post knowledge assessment score.	Higher post-training scores were intended to indicate greater knowledge following simulator-assisted education.	N / N / N	N / N / N	N / N / N	N / N / N	N / N / N	The study demonstrated objective knowledge improvement but did not report sufficient evidence addressing any of the Messick validity domains. All five domains were therefore rated as not assessable.
18	Kantar et al. - stone models	Learner perception ratings comparing 3D stone models with 2D images.	Ratings were intended to reflect learner preference, satisfaction, or perceived educational value of the models rather than objectively measured knowledge or technical skill.	N / N / N	N / N / N	N / N / N	N / N / N	N / N / N	The study compared learner perceptions of 3D stone models and 2D images but did not provide formal validity evidence for assessment scores. Consequently, none of the Messick domains were assessable.
1	Volk et al.	Objective pre/post	Higher post-training	N / N /	N / N /	N / N / N	N / N / N	N / N / N	Although objective pre/post knowledge

9		knowledge assessment score.	scores were intended to indicate increased knowledge following the educational intervention.	N	N				improvement was demonstrated, the study did not report evidence supporting the interpretation of assessment scores within the Messick framework. All five domains were therefore rated as not assessable.
20	Michaels et al.	Pre-course and procedure-specific post-course electronic survey responses.	Survey responses were intended to reflect participants' self-reported expertise, confidence, and/or perceptions before and after the simulation-based course rather than objectively demonstrated technical performance.	N / N / N	1 / 1 / 1	N / N / N	N / N / N	N / N / N	Standardized pre- and post-course electronic survey administration at predefined assessment time points supported limited Response Process evidence (1). No assessable evidence was reported for Content, Internal Structure, Relations to Other Variables, or Consequences.
21	Stanek et al.	Standardized pre/post participant self-assessment responses	Changes in responses were intended to reflect changes in self-perceived confidence or educational benefit, not objectively demonstrated technical competence.	1 / N / N	1 / N / 1	N / N / N	N / N / N	N / N / N	Standardized pre/post self-assessment supported limited Response Process evidence. No assessable evidence was reported for Content, Internal Structure, Relations to Other Variables, or Consequences.
22	Plana et al.	Objective cleft-lip surgical-marking performance score derived from repeated blinded ratings by two expert raters.	Higher scores were intended to indicate more accurate or proficient cleft-lip surgical marking within the simulated setting.	N / 1 / 1	2 / 2 / 2	2 / 2 / 2	N / N / N	N / N / N	Expert-developed procedural content supported limited Content evidence. Blinded repeated scoring by two expert raters supported strong Response Process evidence, while formal intra- and interrater ICC analyses supported strong Internal Structure evidence. No assessable evidence was reported for Relations to Other Variables or Consequences.
2	Kantar et	Cleft-specific	Higher	N / 1 / 1	2 / 2 / 2	2 / 2 / 2	N / N / N	N / N / N	Expert-developed cleft-specific procedural

3	al. - digital simulation	procedural/technical-performance and surgical-marking observations scored by blinded experts; objective knowledge outcomes were also reported.	performance/marking scores were intended to indicate better cleft-specific procedural performance within the digital/simulated educational environment; higher knowledge scores reflected greater knowledge.						content supported limited Content evidence. Blinded expert assessment supported strong Response Process evidence, while strong interrater ICCs supported strong Internal Structure evidence. No assessable evidence was reported for Relations to Other Variables or Consequences.
24	Chang et al.	Pre- and post-course Likert-style survey responses regarding self-reported knowledge/confidence/perceptions.	Changes in survey ratings were intended to reflect participants' self-reported knowledge, confidence, and perceptions following the course rather than objectively graded technical competence.	1 / N / N	1 / N / 1	N / N / N	N / N / N	N / N / N	Pre- and post-course surveys administered at predefined assessment time points supported limited Response Process evidence (1). No assessable evidence was reported for Content, Internal Structure, Relations to Other Variables, or Consequences.

Note. Values are presented as Reviewer 1 rating / Reviewer 2 rating / final consensus rating. The first two entries represent the updated independent assessments performed during minor revision using the final operational criteria. Agreement statistics were calculated from these ratings before consensus adjudication. The third entry represents the final classification after resolution of disagreements. Validity-evidence rating scheme adapted from Beckman et al.: N = relevant evidence was not reported or could not be assessed; 0 = the domain was assessed but findings did not support the intended interpretation; 1 = limited, indirect, partial, or methodologically incomplete supporting evidence; and 2 = strong, direct, and methodologically robust supporting evidence. Domain ratings were considered independently and were not summed.

Table S3. Independent reviewer ratings, final consensus, and classification rationale for McGaghie educational effectiveness

ID	Publication	Reviewer 1	Reviewer 2	Final consensus	Classification rationale
1	Zaga-Galante et al.	1	1	1	Educational outcomes were based on expert/learner perceptions without objective demonstration of knowledge or technical-performance effects.
2	Lutz et al. - bilateral	1	1	1	Educational outcomes were predominantly based on participant self-assessment, without objective performance assessment.
3	Lutz et al. - unilateral	1	1	1	Educational benefit was assessed through self-reported outcomes without objective performance measurement.
4	Vogl et al.	2	2	2	Objective improvement in knowledge and anatomy-related task performance was demonstrated within the simulated setting.
5	Wang et al.	2	2	2	Objective theoretical and operative outcomes favored simulator-assisted education.
6	Riedle et al.	1	1	1	Outcomes focused on feasibility, realism, usability, and perceived educational benefit without objective performance improvement.
7	Kantar et al. - 5-year digital assessment	1	1	1	Outcomes reflected platform use, learner reaction, and perceived usefulness without objective knowledge or performance assessment.
8	Rogers-Vizena et al. - simulator development	Not evaluated	Not evaluated	Not evaluated	The study evaluated simulator development, anatomical fidelity, and procedural characteristics but did not assess learner reaction or learning outcomes. Educational effectiveness was therefore not evaluated.
9	Reighard et al.	1	1	1	Outcomes were primarily developmental and perceptual, without objective demonstration of learner performance effects.
10	Khoo et al.	1	1	1	Improvement was limited to self-reported confidence without objective knowledge or technical-performance assessment.
11	Podolsky et al.	1	1	1	Educational outcomes were based on expert evaluation of realism and utility rather than objective

					learner performance.
12	Saldanha et al.	1	1	1	Educational improvement was predominantly self-reported despite collection of additional anthropometric data.
13	Teuber Lobos et al.	1	1	1	Outcomes focused on perceived realism, usability, and educational value without objective learning or performance assessment.
14	Rogers-Vizena et al. - retention	2	2	2	Objective simulated performance improvement and retention were demonstrated using structured performance measures.
15	Rogers-Vizena et al. - competency	Not evaluated	Not evaluated	Not evaluated	The study evaluated whether simulator-based performance discriminated between training levels. This provided assessment/validity evidence but did not demonstrate learning following an educational intervention or assess learner reaction. Educational effectiveness was therefore not evaluated.
16	Kaimi et al.	2	2	2	Repeated objective technical-performance assessment demonstrated a learning curve and differentiation across experience levels.
17	Chen et al.	2	2	2	Objective improvement in knowledge was demonstrated following simulator-assisted education.
18	Kantar et al. - stone models	1	1	1	Outcomes reflected learner satisfaction and perceived educational value without objective acquisition of technical skill.
19	Volk et al.	2	2	2	Objective pre/post improvement in knowledge was demonstrated.
20	Michaels et al.	1	1	1	Educational effects were based on self-reported expertise and confidence without objective performance assessment.
21	Stanek et al.	1	1	1	Educational benefit and confidence were assessed subjectively, without objective knowledge or technical-performance outcomes.
22	Plana et al.	2	2	2	Objective improvement in cleft lip surgical marking performance was demonstrated.
23	Kantar et al. - digital simulation	2	2	2	Objective improvements in knowledge, surgical marking, and simulated technical performance were demonstrated.

24	Chang et al.	1	1	1	Educational change was based primarily on self-reported expertise and confidence without objective technical grading.

Note. The Reviewer 1 and Reviewer 2 columns present the updated independent classifications assigned during minor revision using the final operational criteria. The final consensus column presents the classifications after adjudication. Percentage agreement and Cohen's κ were calculated from the updated independent classifications before adjudication. Not evaluated indicates that the publication did not assess learner reaction or learning outcomes and is not a McGaghie level. Level 1 denotes learner reaction or subjective educational outcomes. Level 2 requires objectively demonstrated improvement in knowledge or technical performance following an educational intervention or repeated simulation, including retention of objectively measured gains. Performance discrimination based solely on prior training or experience was treated as assessment/validity evidence and was not sufficient for Level 2 classification. Levels 3–5 denote clinical transfer, patient-level benefit, and organizational or healthcare-system effects, respectively. The final distribution was 14 publications at Level 1, eight at Level 2, and two with educational effectiveness not evaluated.
