# Supplementary material for: Simulation-Based Training Tools for Cleft Lip Repair: A Systematic Review
Source: J Clin Med. 2026 Oct 13;15(20):7911. doi: 10.3390/jcm15207911 (PMC13706072; doi:10.3390/jcm15207911)
Supplement: Supplementary file 1 [file jcm-15-07911-s001.zip › Supplementary File S7_ Mixed Methods Appraisal Tool (MMAT) Assessment.pdf]

A. Quantitative randomized controlled trials

Study	Are there clear research questions?	Do the collected data allow to address the research questions?	Is randomization appropriately performed?	Are the groups comparable at baseline?	Are there complete outcome data?	Are outcome assessors blinded to the intervention provided?	Did the participants adhere to the assigned intervention ?	Study-specific rationale
Wang et al. 2025	Yes	Yes	Can’t tell	Yes	Yes	Can’t tell	Yes	The research question was clearly stated, and the collected data were sufficient to address the study objective. Randomization was rated as “Can’t tell” because the authors reported that participants were randomly divided into two groups but did not describe the method used to generate the allocation sequence. Baseline comparability was rated as “Yes” because both groups were drawn from the same educational cohort and had nearly identical baseline cleft lip knowledge scores. Outcome data were considered complete because all 40 participants were represented in the reported post-intervention analyses, with 20 participants in each group. Outcome-assessor blinding was rated as “Can’t tell” because operative performance was assessed by two clinicians, but whether they were blinded to group allocation was not reported; participant-reported questionnaire outcomes were inherently unblinded. Adherence was rated as “Yes” because both groups completed their assigned educational programs under comparable teaching conditions, with no reported crossover, withdrawal, or protocol deviation.
Chen et al. 2024	Yes	Yes	Yes	Yes	Can’t tell	No	Yes	The research question was clearly stated, and the collected data were sufficient to address the study objective. Randomization was rated as “Yes” because random numbers were generated in Microsoft Excel for all 53 participants, sorted in ascending order, and used to allocate the first 26 students to the control group and the remaining 27 to the experimental group. Baseline comparability was rated as “Yes” because no significant differences were reported in age, gender, or year of study, and none of the participants had previous theoretical or practical training in cleft lip repair.

								Completeness of outcome data was rated as “Can’t tell” because 53 participants were reported as enrolled and allocated 26/27 between groups, whereas the outcome tables reported 27 participants in each group; this discrepancy was not explained. Outcome-assessor blinding was rated as “No” because blinding was not reported and participant-reported questionnaire outcomes were inherently unblinded. Adherence was rated as “Yes” because both groups received their assigned educational intervention, delivered independently by the same instructor, with no reported crossover or protocol deviation.
Plana et al. 2019	Yes	Yes	Can’t tell	Yes	Yes	Yes	Yes	The research question was clearly stated, and the collected data were sufficient to address the study objective. Randomization was rated as “Can’t tell” because participants were reported as randomly assigned to the digital simulation or textbook group, but the method used to generate the random allocation sequence was not described. Baseline comparability was rated as “Yes” because participants were drawn from the same population of first-year medical student novice learners and pre-intervention marking scores were comparable between groups. Outcome data were considered complete because all 35 randomized participants were represented in the pre- and post-intervention analyses, with no attrition reported before outcome assessment. Outcome-assessor blinding was rated as “Yes” because two craniofacial surgeons graded the markings while blinded to both group allocation and pre- versus post-intervention status. Adherence was rated as “Yes” because participants completed the assigned 20-minute educational intervention without additional assistance or instruction, and crossover to the alternative resource occurred only after completion of the primary post-intervention assessment.
Kantar et al. 2020	Yes	Yes	Can’t tell	Yes	Yes	Yes	Yes	The research question was clearly stated, and the collected data were sufficient to address the study objective. Randomization was rated as “Can’t tell” because participants were reported as randomized to the digital simulation or textbook group, but the method used to generate the random allocation sequence was not described. Baseline comparability was rated as “Yes” because age, sex, postgraduate year, and previous cleft procedure experience were comparable between groups. Outcome data were

								considered complete because all 13 randomized participants were represented in the pre- and post-intervention analyses, with no reported attrition. Outcome-assessor blinding was rated as "Yes" because markings and surgical performance were independently graded in a blinded fashion by two expert craniofacial surgeons; participant-reported confidence and satisfaction outcomes were inherently unblinded. Adherence was rated as "Yes" because both groups completed standardized exposure to their assigned educational resource, with identical study times and no crossover before completion of the primary post-intervention assessments.

B. Quantitative non-randomized studies

Study	Are there clear research questions?	Do the collected data allow to address the research questions?	Are the participants representative of the target population?	Are measurements appropriate regarding both the outcome and intervention (or exposure)?	Are there complete outcome data?	Are the confounders accounted for in the design and analysis?	During the study period, is the intervention administered (or exposure occurred) as intended?	Study-specific rationale
Vogl et al. 2025	Yes	Yes	Can't tell	Can't tell	Can't tell	No	Yes	The research question was clearly stated, and the collected data were sufficient to address the study objectives. Participant representativeness was rated as "Can't tell" because participants were recruited from specific educational courses and a specialist workshop, while the sampling frame and characteristics of eligible non-participants were not reported. Measurement appropriateness was rated as "Can't tell" because the study combined objective 3D measurements and structured knowledge tasks with study-specific self-assessment questionnaires and an ad hoc three-level assessment of student surgical outcomes for which formal validity evidence was not reported. Completeness of outcome data was rated as "Can't tell" because ten specialists participated in the unilateral simulation whereas only eight were reported for the bilateral

								simulation, despite the protocol indicating that participants received both models, and the reason for the missing bilateral observations was not provided. Confounding was rated as “No” because participant experience varied substantially and comparisons between different course formats were not adjusted for potential confounding factors. Intervention administration was rated as “Yes” because the courses followed predefined instructional protocols and the planned ex vivo models and step-by-step training were delivered without reported protocol deviations.
Khoo et al. 2023	Yes	Yes	Yes	Can’t tell	Yes	No	Yes	The research question was clearly stated, and the collected data were sufficient to assess changes in perceived procedural confidence following simulation training. Participant representativeness was rated as “Yes” because 11 of the 12 plastic surgery trainees in Western Australia participated, representing nearly the entire relevant trainee population in the study setting. Measurement appropriateness was rated as “Can’t tell” because the confidence questionnaire was adapted from a validated procedural confidence instrument, but validation of the cleft-specific adapted version was not reported and the outcomes were self-reported rather than objective measures of technical performance. Outcome data were considered complete because all 11 participants were represented in the pre- and post-simulation analyses, with no reported attrition. Confounding was rated as “No” because participants differed in training level and previous cleft lip experience, and these potential confounders were not formally adjusted for in the analysis. Intervention administration was rated as “Yes” because the workshop followed a predefined format using high-fidelity simulation models, instructional video, and teaching by two senior cleft surgeons, with no reported protocol deviations.
Saldanha et al. 2021	Yes	Yes	Can’t tell	Yes	Yes	No	Yes	The research question was clearly stated, and the collected data were sufficient to address the educational objectives. Participant representativeness was rated as “Can’t tell” because 16 residents and fellows from several training tracks participated, but the eligible source population, recruitment process, participation rate, and characteristics of

								non-participants were not reported. Measurement appropriateness was rated as “Yes” because the self-assessment instrument was developed with input from a survey-methodology statistician and referenced the Plastic Surgery Milestones, a validated self-confidence questionnaire was used, and objective surgical outcomes were assessed using standardized three-dimensional anthropometric measurements. Outcome data were considered complete because all 16 participants completed the curriculum and were represented in the reported analyses. Confounding was rated as “No” because, although training level and prior surgical experience were explored, the uncontrolled pre-post design did not formally control for potential confounding of the educational effect, and the effects of instructional video and simulation could not be separated. Intervention administration was rated as “Yes” because the curriculum followed a predefined sequence of lecture, instructional video, and hands-on simulation, with no reported protocol deviations.
Rogers-Vizena et al. 2023	Yes	Yes	Can’t tell	Yes	Can’t tell	Yes	Yes	The research question was clearly stated, and the collected data were sufficient to assess both immediate educational improvement and retention over time. Participant representativeness was rated as “Can’t tell” because participation was voluntary and the number and characteristics of eligible non-participants were not reported. Measurement appropriateness was rated as “Yes” because objective performance was assessed using blinded video-based modified OSATS and the procedure-specific UCLR tool, subjective outcomes were measured using previously published or validated instruments, and three-dimensional symmetry assessment was used to support the UCLR scoring. Completeness of outcome data was rated as “Can’t tell” because 21 of 26 participants returned for the approximately 3-month follow-up; although some attrition occurred during a COVID-related interruption, the characteristics of participants lost to follow-up and explicit handling of missing follow-up data were not reported. Confounding was rated as “Yes” because the repeated-measures MANOVA adjusted for training level, time between follow-up sessions, and intervening cleft lip repair experience. Intervention administration was rated as “Yes” because

								orientation, simulation procedures, surgeon assistance, and structured debriefing were standardized across participants.
Rogers-Vizena et al. 2022	Yes	Yes	Can’t tell	Yes	Yes	No	Yes	The research question was clearly stated, and the collected data were sufficient to evaluate whether high-fidelity simulation could discriminate cleft lip repair performance across levels of training. Participant representativeness was rated as “Can’t tell” because residents and fellows were recruited from several training programs, but participation was voluntary and the number and characteristics of eligible non-participants were not reported. Measurement appropriateness was rated as “Yes” because performance was independently and blindly assessed by two practicing cleft surgeons using modified OSATS and the procedure-specific UCLR tool, supplemented by objective three-dimensional symmetry measurements. Outcome data were considered complete because all 26 participants were represented in the primary analyses; although one participant did not fully complete the simulated repair, no participant-level loss to primary outcome assessment was reported. Confounding was rated as “No” because training level and cumulative prior experience were examined separately using bivariate correlations without multivariable adjustment for potential confounding. Intervention administration was rated as “Yes” because orientation, simulator use, and surgical assistance were standardized across participants, with no coaching provided during the procedure.
Kaimi et al. 2025	Yes	Yes	Can’t tell	Yes	Can’t tell	No	Yes	The research question was clearly stated, and the collected data were sufficient to evaluate the technical assessment tool and changes in simulated performance across repeated sessions. Participant representativeness was rated as “Can’t tell” because participants were recruited from a single institution and the eligible source population and characteristics of non-participants were not reported. Measurement appropriateness was rated as “Yes” because the cleft lip-specific assessment scale was developed using a structured nominal group process involving expert cleft surgeons, a previously validated global rating scale was also used, and three calibrated expert raters assessed randomized, coded video recordings while blinded to participant identity.

								Completeness of outcome data was rated as “Can’t tell” because 33 procedures would be expected from the reported study protocol, whereas only 31 videos were reported as assessed, and the reason for the two missing recordings was not provided. Confounding was rated as “No” because participants differed substantially in previous cleft lip experience and training background, and these factors were not controlled using adjusted multivariable analyses. Intervention administration was rated as “Yes” because trainees received standardized preparatory teaching, repeated simulator sessions, and structured feedback before subsequent sessions, with no reported systematic protocol deviations.
Kantar et al. 2019	Yes	Yes	Can’t tell	Can’t tell	Yes	No	Yes	The research question was clearly stated, and the collected data were sufficient to compare learner satisfaction with the two educational tools. Participant representativeness was rated as “Can’t tell” because 13 plastic surgery residents were prospectively recruited, but the eligible source population, recruitment fraction, selection process, and characteristics of non-participants were not reported. Measurement appropriateness was rated as “Can’t tell” because learner satisfaction was assessed using a modified questionnaire based on the validated Student Evaluation of Educational Quality instrument, but the psychometric properties of the modified version used in this study were not reported. Outcome data were considered complete because all 13 participants were represented in the reported analyses, with no attrition or missing participant-level outcomes described. Confounding was rated as “No” because all participants evaluated the stone model before the standardized photograph, without randomization or counterbalancing of exposure order, and potential order effects were not controlled analytically. Intervention administration was rated as “Yes” because participants received standardized preparatory materials, study time, and exposure to both educational tools, with no reported protocol deviations.
Volk et al. 2020	Yes	Yes	Can’t tell	Can’t tell	Yes	No	Yes	The research question was clearly stated, and the collected data were sufficient to evaluate changes in trainee knowledge and confidence after use of the virtual simulator. Participant representativeness was rated as

								“Can’t tell” because trainees from a single residency program were invited, but the total eligible population, participation rate, and characteristics of non-participants were not reported. Measurement appropriateness was rated as “Can’t tell” because knowledge was assessed using a study-specific 16-item test and confidence using an explicitly nonvalidated questionnaire, without reported validity or reliability assessment for these measures. Outcome data were considered complete because all 16 participants were represented in the reported pre- and post-intervention analyses, with no reported attrition. Confounding was rated as “No” because the uncontrolled pre-post design did not adjust for training level, previous cleft exposure, or other potential confounding factors. Intervention administration was rated as “Yes” because all participants completed the same three predefined simulator modules before post-intervention assessment, with no reported protocol deviations.
Stanek et al. 2025	Yes	Yes	Can’t tell	Yes	Yes	No	Yes	The research question was clearly stated, and the collected data were sufficient to compare perceived educational improvement between lower- and higher-complexity cleft lip simulators. Participant representativeness was rated as “Can’t tell” because residents and fellows from relevant training programs were eligible, but participation was voluntary and the total eligible population and characteristics of non-participants were not reported. Measurement appropriateness was rated as “Yes” because cleft-specific knowledge and skill were assessed using a previously used self-assessment instrument and procedural confidence using a previously validated questionnaire, consistent with the study’s stated focus on perceived educational value. Outcome data were considered complete because all 52 included participants were represented in the comparative analyses, with 26 participants in each simulator group and no reported attrition. Confounding was rated as “No” because simulator allocation was primarily chronological, the HCS group combined retrospective and prospective participants, and baseline presimulation scores differed between groups; although the analysis adjusted for PGY and included a sensitivity analysis, residual

								confounding related to cohort and baseline differences could not be excluded. Intervention administration was rated as “Yes” because both groups received the same structured bootcamp-style educational program led by the same cleft surgeon, with no reported relevant protocol deviations.

C. Quantitative descriptive studies

Study	Are there clear research questio ns?	Do the collected data allow to address the research questions?	Is the sampling strategy relevant to address the research question?	Is the sample representative of the target population?	Are the measurements appropriate?	Is the risk of nonresponse bias low?	Is the statistical analysis appropriate to answer the research question?	Study-specific rationale
Zaga-Galante et al. 2025	Yes	Yes	Yes	No	Yes	Yes	Yes	The research question was clearly stated, and the collected data were sufficient to evaluate the simulator’s anatomical accuracy, realism, and perceived educational value. The sampling strategy was rated as “Yes” because experienced cleft surgeons were an appropriate expert population for assessing these characteristics. Sample representativeness was rated as “No” because only 11 surgeons were recruited according to their availability at a single craniofacial meeting, constituting a convenience sample that was not representative of the broader target population. Measurement appropriateness was rated as “Yes” because a structured Likert-type questionnaire developed by cleft lip experts directly assessed the intended constructs and was completed by experienced surgeons who were not involved in simulator development. Risk of nonresponse bias was rated as low because all 11 recruited surgeons completed the simulated repair and provided questionnaire evaluations. Statistical analysis was rated as appropriate because the study was a preliminary descriptive evaluation and questionnaire responses were appropriately summarized using frequencies and mean Likert scores.

Lutz et al. 2023 (bilateral)	Yes	Yes	Can’t tell	Can’t tell	No	Yes	Yes	The research question was clearly stated, and the collected data were sufficient to provide a preliminary evaluation of the ex vivo bilateral cleft lip training model. Sampling strategy was rated as “Can’t tell” because 12 participants attended the course, but the recruitment process, eligibility framework, and rationale for participant selection were not sufficiently described. Sample representativeness was rated as “Can’t tell” because participants had limited previous cleft lip experience, but the source population, participation rate, and characteristics of non-participants were not reported. Measurement appropriateness was rated as “No” because educational outcomes were assessed using a self-designed, nonvalidated self-assessment questionnaire and no objective assessment of technical performance was performed. Risk of nonresponse bias was rated as low because all 12 participant questionnaires were evaluated. Statistical analysis was rated as appropriate because the study was a preliminary descriptive evaluation and questionnaire responses were summarized using descriptive statistics consistent with the study objective.
Lutz et al. 2023 (unilateral)	Yes	Yes	Can’t tell	Can’t tell	No	Yes	Yes	The research question was clearly stated, and the collected data were sufficient to provide a preliminary evaluation of the unilateral cleft lip training model. Sampling strategy was rated as “Can’t tell” because 16 young medical professionals participated, but the recruitment procedure, sampling frame, eligibility criteria, and rationale for participant selection were not sufficiently described. Sample representativeness was rated as “Can’t tell” because participants included CMF residents, oral surgery residents, and licensed physicians or dentists interested in CMF training with limited previous cleft experience, while the underlying eligible population, participation rate, and characteristics of non-participants were not reported. Measurement appropriateness was rated as “No” because educational outcomes were assessed using a self-designed questionnaire that had not been validated, as explicitly acknowledged by the authors. Risk of nonresponse bias was rated as low because results were reported for the full group of 16 participants, with no reported questionnaire nonresponse or missing participant-level outcomes.

								Statistical analysis was rated as appropriate because the study was a preliminary descriptive evaluation and questionnaire responses were summarized using standard descriptive statistics.
Riedle et al. 2019	Yes	Yes	Can’t tell	Can’t tell	No	Can’t tell	Yes	The research question was clearly stated, and the collected data were sufficient to provide a preliminary evaluation of the 3D-printed silicone cleft lip and palate model. Sampling strategy was rated as “Can’t tell” because surgical residents from two institutions evaluated the model, but the recruitment strategy, eligibility criteria, sampling frame, and rationale for participant selection were not sufficiently described. Sample representativeness was rated as “Can’t tell” because 20 residents provided evaluation data, while the underlying eligible population and characteristics of non-participants were not reported. Measurement appropriateness was rated as “No” because outcomes were assessed using a study-specific questionnaire without reported validation or reliability testing, and perceived improvement in surgical technique was based on participant self-assessment. Risk of nonresponse bias was rated as “Can’t tell” because 30 models were distributed but evaluation data were reported for only 20 residents, without explanation of the discrepancy or information about nonresponders. Statistical analysis was rated as appropriate because the study was primarily descriptive and questionnaire responses were summarized using descriptive statistics consistent with the study objective.
Kantar et al. 2018	Yes	Yes	Yes	Can’t tell	Yes	Can’t tell	Yes	The research question was clearly stated, and the collected data were sufficient to evaluate simulator demographics, usage, global reach, and perceived utility. Sampling strategy was rated as “Yes” because objective platform data were collected directly from simulator users and surveys were distributed to registered users and Smile Train partners, populations relevant to the study objectives. Sample representativeness was rated as “Can’t tell” because, although 132 simulator users and 42 Smile Train partners completed the surveys, the total number invited and characteristics of nonrespondents were not reported. Measurement appropriateness was rated as “Yes” because simulator usage was objectively recorded through an embedded analytics database using

								predefined usage metrics, while survey questions directly addressed user characteristics and perceived simulator utility. Risk of nonresponse bias was rated as "Can't tell" because survey response rates and characteristics of nonrespondents could not be determined. Statistical analysis was rated as appropriate because the study was descriptive and the collected usage and survey data were summarized using appropriate descriptive statistics.
Rogers-Vizena et al. 2018	Yes	Yes	Yes	Can't tell	Yes	Yes	Yes	The research question was clearly stated, and the collected data were sufficient to provide a preliminary evaluation of the high-fidelity cleft lip simulator. Sampling strategy was rated as "Yes" because experienced cleft surgeons were an appropriate expert population for assessing whether the simulator reproduced relevant anatomy, haptics, and procedural steps. Sample representativeness was rated as "Can't tell" because only three surgeons participated, including one study author, and the selection process and broader eligible expert population were not described. Measurement appropriateness was rated as "Yes" because simulator performance was evaluated using three-dimensional scans, standard anatomical landmarks, indirect anthropometry, surface-change mapping, and comparison with real-patient data. Risk of nonresponse bias was rated as low because all three participating surgeons contributed evaluation data, with no reported missing assessments. Statistical analysis was rated as appropriate because this was a small pilot validation study and descriptive anthropometric and three-dimensional comparisons were used, while formal inferential comparison was appropriately avoided due to the limited sample and substantial individual variation.
Reighard et al. 2019	Yes	Yes	Can't tell	Can't tell	Yes	Yes	Yes	The research question was clearly stated, and the collected data were sufficient to provide a preliminary evaluation of the low-cost high-fidelity cleft lip simulator. Sampling strategy was rated as "Can't tell" because experienced cleft surgeons were an appropriate expert population, but the method used to identify and select the five participating surgeons was not sufficiently described. Sample representativeness was rated as "Can't tell" because only five surgeons

								from three academic institutions participated and the broader eligible population and characteristics of non-participants were not reported. Measurement appropriateness was rated as “Yes” because the 22-item survey was developed from a cognitive task analysis, independently reviewed by three cleft surgeons for clarity and alignment with clinical practice and learning objectives, and evaluated using previously validated Standards-based methods. Risk of nonresponse bias was rated as low because all five participating surgeons contributed ratings, with no reported missing participant-level evaluations. Statistical analysis was rated as appropriate because this was a preliminary descriptive validation study and expert ratings were appropriately summarized using means, measures of variability, and confidence intervals.
Podolsky et al. 2018	Yes	Yes	Yes	Can’t tell	No	Yes	Yes	The research question was clearly stated, and the collected data were sufficient to provide a preliminary evaluation of the high-fidelity cleft lip simulator. Sampling strategy was rated as “Yes” because four expert cleft surgeons with more than 100 previous cleft lip repairs each were an appropriate population for assessing simulator anatomy, procedural realism, and training value. Sample representativeness was rated as “Can’t tell” because only four experts participated and the method used to select them from the broader population of experienced cleft surgeons was not reported. Measurement appropriateness was rated as “No” because simulator evaluation relied on qualitative surgeon feedback without a reported structured or validated assessment instrument or reliability assessment. Risk of nonresponse bias was rated as low because all participating surgeons completed the simulated procedures and contributed feedback. Statistical analysis was rated as appropriate because this was a small preliminary simulator evaluation and the findings were reported descriptively without unsupported inferential comparisons.
Teuber Lobos et al. 2024	Yes	Yes	Can’t tell	Can’t tell	Can’t tell	Yes	Yes	The research question was clearly stated, and the collected data were sufficient to provide a preliminary evaluation of the open-source cleft lip and palate simulator. Sampling strategy was rated as “Can’t tell” because trainees and experienced maxillofacial surgeons were relevant

								populations for simulator evaluation, but the recruitment procedure, sampling frame, and rationale for participant selection were not sufficiently described. Sample representativeness was rated as "Can't tell" because participants were recruited from two centers, while the underlying eligible population, participation rate, and characteristics of non-participants were not reported. Measurement appropriateness was rated as "Can't tell" because the questionnaire directly assessed predefined face- and content-validity domains using clearly specified Likert items, but no formal validation or reliability assessment of the study-specific instrument was reported. Risk of nonresponse bias was rated as low because all 16 participants responded to the questionnaire following simulator testing. Statistical analysis was rated as appropriate because descriptive statistics were used to summarize the ordinal ratings and the Wilcoxon rank-sum test was used for comparisons between the independent trainee and surgeon groups.

D. Mixed methods studies - Screening questions*

Study	Are there clear research questions?	Do the collected data allow to address the research questions?	Study-specific rationale
Michaels et al. 2022	Yes	Yes	The research questions were clearly stated, with the study aiming both to quantify the effect of the virtual simulation course on fellows' perceived procedural expertise and confidence and to explore their perspectives on the value of the telesimulation format. The collected data were sufficient to address these objectives because the study combined pre- and post-course quantitative surveys with free-text qualitative feedback concerning participants' experiences and perceptions of the course.
Chang et al. 2020	Yes	Yes	The research questions were clearly stated, with the study aiming to quantify the effects of a multistation simulation curriculum on trainees' self-reported expertise and confidence and to evaluate their perceptions of the educational experience. The collected data were sufficient to address these objectives because pre- and post-course surveys incorporated quantitative ratings of expertise, confidence, and attitudes as well as qualitative narrative feedback regarding the simulators and course structure.

D1. Qualitative component of mixed methods studies

Study	Is the qualitative approach appropriate to answer the research question?	Are the qualitative data collection methods adequate to address the research question?	Are the findings adequately derived from the data?	Is the interpretation of results sufficiently substantiated by data?	Is there coherence between qualitative data sources, collection, analysis and interpretation?	Study-specific rationale
Michaels et al. 2022	Yes	Yes	Can't tell	Yes	Can't tell	The qualitative approach was considered appropriate because free-response survey questions were used to explore fellows' experiences, perceptions, and feedback regarding the virtual simulation course. Qualitative data collection was adequate because the procedure-specific and general post-course surveys included multiple open-ended questions addressing the educational value, structure, and implementation of the course. Derivation of the findings was rated as "Can't tell" because the authors reported using thematic text analysis but did not describe the coding process, number of reviewers, development of themes, or procedures for resolving differences in interpretation. Interpretation was considered sufficiently substantiated because the reported themes were supported by representative participant quotations concerning hands-on experience, course structure, and logistical advantages. Coherence between qualitative data sources, collection, analysis, and interpretation was rated as "Can't tell" because the limited description of the thematic analytic process prevented full assessment of how the original free-text responses were transformed into the reported themes.
Chang et al. 2020	Yes	Yes	Can't tell	Yes	Can't tell	The qualitative approach was considered appropriate because narrative feedback was collected to explore trainees' experiences with the simulators and the structure of the simulation course. Qualitative data collection was adequate because the post-course surveys provided opportunities for participants to give narrative feedback regarding the individual simulators and the overall educational experience. Derivation of the findings was rated as "Can't tell" because the authors reported thematic text analysis but did not describe the coding procedure, number of reviewers, development of themes, or methods for resolving interpretive differences. Interpretation was considered sufficiently substantiated because the reported narrative findings were linked to

						recurrent participant feedback, particularly the desire for additional practice time and the perceived value of exposure to otherwise infrequently encountered procedures. Coherence between qualitative data sources, collection, analysis, and interpretation was rated as “Can’t tell” because insufficient detail regarding the thematic analysis process prevented full assessment of how the raw narrative responses were transformed into the reported themes.

D2. Quantitative non-randomized component of mixed methods studies

Study	Are the participants representative of the target population?	Are measurements appropriate regarding both the outcome and intervention (or exposure)?	Are there complete outcome data?	Are the confounders accounted for in the design and analysis?	During the study period, is the intervention administered (or exposure occurred) as intended?	Study-specific rationale
Michaels et al. 2022	Can’t tell	Can’t tell	Can’t tell	No	Yes	Participant representativeness was rated as “Can’t tell” because 35 pediatric otolaryngology fellows from multiple residency and fellowship programs participated, but the total eligible population, participation rate, and characteristics of non-participants were not reported. Measurement appropriateness was rated as “Can’t tell” because procedural expertise, confidence, and perceptions of the course were assessed using study-specific Likert-scale surveys, without reported validation or reliability assessment of these instruments. Completeness of outcome data was rated as “Can’t tell” because the manuscript did not clearly report complete pre- and post-course survey denominators for all analyzed outcomes, preventing a definitive assessment of outcome-data completeness. Confounding was rated as “No” because the uncontrolled pre-post design did not adjust for differences in prior procedural experience or other potential confounders, and the intervention combined pre-course educational materials, simulator practice, live expert instruction, and feedback, preventing attribution of the observed changes to an individual component. Intervention administration was rated as “Yes” because the planned four-station virtual curriculum was delivered using standardized simulator materials, instructional resources, expert-led breakout rooms, and predefined station times, with no major technical difficulties reported.

Chang et al. 2020	Can't tell	Can't tell	Yes	No	Yes	Participant representativeness was rated as "Can't tell" because 9 pediatric otolaryngology fellows and 17 otolaryngology residents from international institutions were recruited, but the total eligible population, participation rate, and characteristics of non-participants were not reported. Measurement appropriateness was rated as "Can't tell" because expertise, confidence, and attitudes were assessed using study-specific pre- and post-course Likert-based surveys without a previously validated instrument, as explicitly acknowledged by the authors. Completeness of outcome data was rated as "Yes" because 26 pre-course surveys and 25 post-course surveys were returned, corresponding to a 96% response rate and minimal loss of outcome data. Confounding was rated as "No" because the quasi-experimental uncontrolled pre-post design included participants with heterogeneous training levels and prior procedural exposure, and these potential confounders were not formally adjusted for in the primary analysis. Intervention administration was rated as "Yes" because trainees received standardized preparatory materials and rotated through predefined 45-minute simulation stations with two attending surgeons per station, with no reported systematic deviation from the planned course.

D3. Mixed methods component

Study	Is there an adequate rationale for using a mixed methods design to address the research question?	Are the different components of the study effectively integrated to answer the research question?	Are the outputs of the integration of qualitative and quantitative components adequately interpreted?	Are divergences and inconsistencies between quantitative and qualitative results adequately addressed?	Do the different components of the study adhere to the quality criteria of each tradition of the methods involved?	Study-specific rationale
Michaels et al. 2022	Yes	Yes	Yes	Yes	No	The rationale for using both quantitative and qualitative data was considered adequate because the study evaluated changes in fellows' perceived procedural expertise and confidence while also exploring their experiences and perceptions of the virtual course. Meaningful integration occurred primarily at the interpretation stage: quantitative pre/post survey findings on expertise, confidence, and attitudes were considered alongside themes

						derived from free-text responses concerning hands-on experience, course structure, simulator quality, accessibility, and areas for improvement. The Discussion used these complementary findings together to characterize both the educational effect and the practical value of the course. No substantive divergence between the quantitative and qualitative findings was identified; rather, the qualitative findings generally supported the favorable quantitative ratings while providing additional information on implementation and areas requiring improvement. The final criterion was rated as "No" because the individual qualitative and quantitative components did not fully meet all relevant methodological quality criteria, particularly regarding qualitative analytic reporting, measurement validity, completeness of outcome data, and control of confounding.
Chang et al. 2020	Yes	Yes	Yes	Yes	No	The rationale for using both quantitative and qualitative data was considered adequate because the study assessed changes in trainees' self-reported expertise, confidence, and attitudes while also collecting narrative feedback on the simulators and course experience. Meaningful integration occurred primarily at the interpretation stage: the statistically significant pre/post changes in expertise and confidence were discussed together with thematic findings from narrative responses, including trainees' perceived value of exposure to infrequently encountered procedures, requests for additional simulator time, and specific limitations of individual models. The qualitative findings therefore complemented the quantitative results by providing explanatory and implementation-related context. No substantive contradiction between the two components was identified. The final criterion was rated as "No" because the individual qualitative and quantitative components did not fully satisfy all relevant methodological quality criteria, particularly regarding limited reporting of qualitative analysis, uncertain measurement validity, and uncontrolled confounding in the quantitative component.

*For mixed methods studies, the qualitative and quantitative components were appraised separately using the corresponding MMAT criteria, followed by assessment of the mixed methods-specific criteria
